# Monitoring of plant water uptake by measuring root dielectric properties on a fine timescale: diurnal changes and response to leaf excision

**DOI:** 10.1186/s13007-023-01133-8

**Published:** 2024-01-09

**Authors:** Imre Cseresnyés, Anna Füzy, Sándor Kabos, Bettina Kelemen, Kálmán Rajkai, Tünde Takács

**Affiliations:** 1 Institute for Soil Sciences, HUN-REN Centre for Agricultural Research, Herman Ottó út 15., 1022 Budapest, Hungary; 2 Department of Statistics, Eötvös Loránd University, Pázmány Péter stny. 1/A., 1117 Budapest, Hungary

**Keywords:** Dielectric monitoring, Dissipation factor, Electrical capacitance, Electrical conductance, Impedance response, Root activity, Root–substrate system, Transpiration

## Abstract

**Background:**

The measurement of root dielectric response is a useful non-destructive method to evaluate root growth and function. Previous studies tracked root development throughout the plant growing cycle by single-time electrical measurements taken repeatedly. However, it is known that root conductivity and uptake activity can change rapidly, coupled with the day/night cycles of photosynthetic and transpiration rate. Therefore, the low-frequency dielectric monitoring of intact root–substrate systems at minute-scale temporal resolution was tested using a customized impedance measurement system in a laboratory environment. Electrical capacitance (C_R_) and conductance (G_R_) and the dissipation factor (D_R_) were detected for 144 h in potted maize, cucumber and pea grown under various light/dark and temperature conditions, or subjected to progressive leaf excision or decapitation. Photosynthetic parameters and stomatal conductance were also measured to evaluate the stress response.

**Results:**

The C_R_ and G_R_ data series showed significant 24-h seasonality associated with the light/dark and temperature cycles applied. This was attributed to the diurnal patterns in whole-plant transpiration (detected via stomatal conductance), which is strongly linked to the root water uptake rate. C_R_ and G_R_ decreased during the 6-day dark treatment, and dropped proportionally with increasing defoliation levels, likely due to the loss of canopy transpiration caused by dark-induced senescence or removal of leaves. D_R_ showed a decreasing trend for plants exposed to 6-day darkness, whereas it was increased markedly by decapitation, indicating altered root membrane structure and permeability, and a modified ratio of apoplastic to cell-to-cell water and current pathways.

**Conclusions:**

Dynamic, in situ impedance measurement of the intact root system was an efficient way of following integrated root water uptake, including diurnal cycles, and stress-induced changes. It was also demonstrated that the dielectric response mainly originated from root tissue polarization and current conduction, and was influenced by the actual physiological activity of the root system. Dielectric measurement on fine timescale, as a diagnostic tool for monitoring root physiological status and environmental response, deserves future attention.

**Supplementary Information:**

The online version contains supplementary material available at 10.1186/s13007-023-01133-8.

## Background

Electrical capacitance measurement in root–substrate systems is a promising, rapid method to indirectly assess root system size (RSS; Table 1) non-destructively [1, 2]. The root capacitance (C_R_) detected between a ground and a plant electrode was linearly correlated with RSS when the same species were grown in the same soil with the same moisture level, and were measured using the same electrode type and placement [3, 4].

**Table 1:** Nomenclature

Symbol	Meaning
A	Capacitor plate area
AC	Alternating current
C	Electrical capacitance
C*	Complex electrical capacitance
CCI	Chlorophyll concentration index
C_R_	Electrical capacitance of root–substrate system
d	Capacitor plate separation
D	Dissipation factor
D_R_	Dissipation factor of root–substrate system
F_v_/F_m_	Maximum quantum yield of PSII photochemistry
g_s_	Stomatal conductance
G	Electrical conductance
G_R_	Electrical conductance of root–substrate system
i	Imaginary unit
L_R_	Root hydraulic conductance
rELA	Ratio of excised leaf area
R	Electrical resistance
RSS	Root system size
SWC	Substrate water content
TLA	Total leaf area
ε_0_	Permittivity of free space
ε_r_	Relative permittivity
ε_r_'	Real part of permittivity
ε_r_"	Imaginary part of permittivity
ε_r_*	Complex relative permittivity
Φ	Impedance phase angle
ω	Angular frequency

**Fig. 1:**
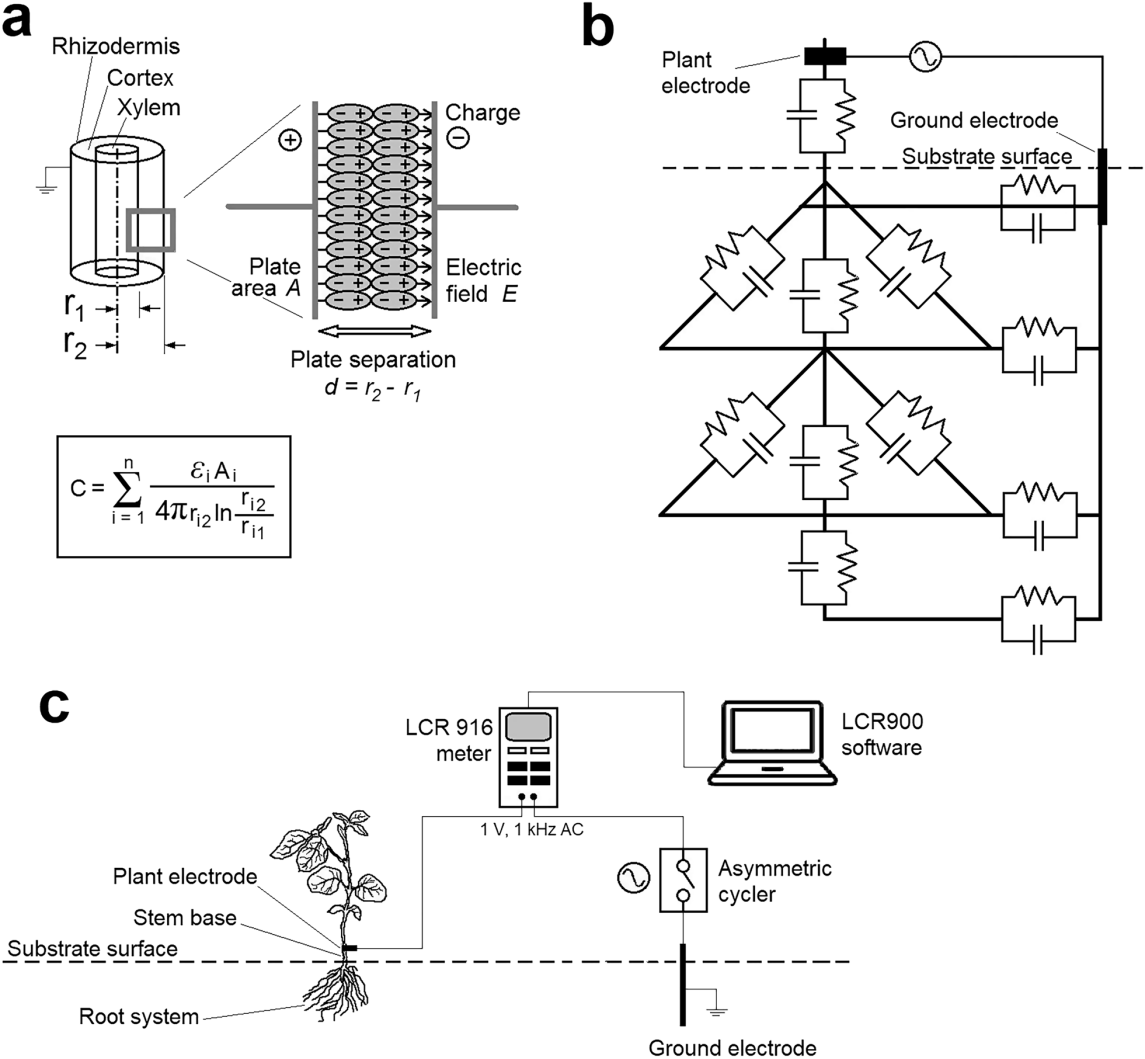
**a** Representation of plant root as a cylindrical capacitor, according to Dalton [6] and Ellis et al. [13]. In the equation for the electrical capacitance C, *ε* and A are the permittivity and surface area of the root tissue, respectively, r_1_ is the xylem radius and r_2_ is the rhizodermis radius. **b** Electrical equivalent network of the root system, according to Dietrich et al. [9] and Ehosioke et al. [1]. **c** Scheme for root dielectric measurement on a fine timescale with a software-controlled LCR meter and an asymmetric cycler, as used in the present study

If a low-frequency alternating current (AC) is driven through a plant tissue, the polarization of membranes and apoplastic and symplastic compartments promotes changes in the amplitude and phase of the AC signal, generating an impedance response [5]. According to the first conceptual model [6], roots are equivalent to cylindrical capacitors, in which the root sap and the soil solution are the inner and outer capacitor plates, respectively, and the membrane acts as a separating dielectric layer (Fig. 1a). The resultant capacitance is directly proportional to the area (A) and the relative permittivity (*ε*_r_) of the charge-storing membranes and inversely proportional to plate separation (d). In physical capacitors, energy is stored electrostatically with an infinitesimal effective energy loss. Living tissues, however, including roots, are leaky capacitors, *i.e.* parallel resistance–capacitance (RC) circuits [7]. The membrane structures are the capacitive elements, whereas the symplast and the water-filled intercellular spaces represent the resistive components [8]. The lossy dielectrics can be characterized by a complex relative permittivity, expressed as: *ε*_r_* = *ε*_r_ˊ– i × *ε*_r_˝, where *ε*_r_ˊ is the real part (energy storage) and *ε*_r_˝ is the imaginary part of permittivity (conductive energy dissipation), and i is the imaginary unit (i^2^ = –1). Thus, a complex capacitance can be defined as: C* = *ε*_0_ × (*ε*_r_ˊ– i × *ε*_r_˝) × A × d^–1^, where *ε*_0_ is the permittivity of free space (8.854 F m^–1^). The ratio of dielectric losses to energy storage is the dissipation factor: D = *ε*_r_˝/*ε*_r_ˊ = G/(*ω* × C), where G is the electrical conductance (= 1/R) and *ω* is the angular frequency. D is a complement to the phase angle (*Φ*) of the capacitive impedance: D = tan(90°– *Φ*).

Later Dietrich et al. [9] questioned the efficiency of the capacitance method and formulated an alternative model (Fig. 1b). They found that roots made a negligible contribution to C_R_, which was dominated by the stem portion between the substrate surface and the plant electrode, proportionally to the stem cross-sectional area. The linear correlations observed between C_R_ and RSS traits were attributed merely to root–shoot allometry. Other studies have shown appreciable current leakage in the root neck and proximal root segments [10, 11]. Conversely, there is experimental evidence to show that most of the root system may be electrically connected, and C_R_ chiefly depends on roots immersed in the growing medium [12, 13]. This is in agreement with previous findings that C_R_ is determined by the root system and is affected but not dominated by the stem base [14]. Furthermore, it was verified that AC could penetrate deep into the root system, especially when the surrounding surface soil was relatively dry and thus less conductive than the roots [15, 16].

There is a general consensus that root histological properties, including water content, tissue density and lignification, regulate the proportion of apoplastic to symplastic current pathways, and modify the impedance response accordingly [6, 11, 13]. This implies an advantage of the dielectric method: the magnitude of C_R_ depends on the macro-scale RSS and the physiological status, which together characterize the uptake intensity of the entire root system. Both during root aging and under stress, both accelerated senescence and exo- and endodermal maturation associated with enhanced suberin deposition, and root decay lead to a decrease in root *ε*_r_ and thus the C_R_ value measured [13]. Moreover, the stress-induced physico-chemical changes in root membranes, including altered surface charge density, impaired structural integrity and enhanced permeability may increase the electrical conductance of the whole root system (G_R_) by increasing root dielectric loss, D_R_ [17–19]. Various abiotic and biotic impacts on the shoot (*e.g.* excess light, extreme temperature, wounding, touching, pressure, insect attack, pathogen injury) also induce electrical signals, resulting in adaptive physiological changes at the whole-plant level [20, 21]. The generation and propagation of stress signals, including action, variation and system potential, are associated with changes in the activity of membrane ion channels and H^+^-ATPase, leading to altered ion fluxes and membrane potential [22, 23]. Although there is no evidence, long-distance electrical signals can be transmitted into the intact parts of the plant, potentially interacting with root impedance response.

As AC flows in the root–substrate continuum by means of ion fluxes (electron conductivity is negligible), predominantly through the water absorption zones and to a much lower extent through the impermeable, suberized surfaces, G_R_ is related to root hydraulic conductance, L_R_ [8, 24]. For the above reasons, the dielectric characterization (measuring C_R_, D_R_ and G_R_ variables) of an intact root–substrate system could be conducive to obtaining information on root extension, physiological status and stress-related changes [2, 25].

On the one hand, some previous studies highlighted the potential of monitoring the impedance response for tracking root growth and function throughout the growing cycle (ontogeny) of plants [6, 12, 26, 27]. These works were based on single-time electrical measurements taken repeatedly on the same potted plants every 3–7 days, or were made in field plant populations every 10–21 days. On the other hand, Weigand and Kemna [24] observed marked diurnal patterns in the apparent conductivity and spectral polarization signatures of oilseed plant roots in hydroponics using electrical impedance spectroscopy (EIS). It is well documented that root systems exhibit clear diurnal variations in L_R_ and solute uptake activity, tied to the light/dark and temperature cycles and to stress-induced changes in shoot functions, such as stomatal conductance (g_s_), photosynthesis and transpiration rate [28]. The oscillations in root hydraulic properties are also coincident with the circadian regulation of the density and water permeability of aquaporin channels located in root cell membranes [29].

Nevertheless, to date, no study has been conducted to test the usability of the single-frequency, two-terminal (one ground and one plant electrode) measurement of dielectric variables (C_R_, D_R_ and G_R_) in intact root–substrate systems at high, *i.e*. minute-scale temporal resolution. Based on previous experience it was hypothesized that, in the frame of a growth chamber study, this novel approach is capable of: (*i*) dynamically measuring the root water uptake activity, including diurnal changes; (*ii*) examining the impact of a modified temperature regime and abnormal photoperiod on the root dielectric response in relation to altered whole-plant transpiration (based on measuring g_s_) and, in certain cases, to the deterioration of the photosynthetic apparatus; and (*iii*) detecting the changes in root uptake rate associated with the reduced total transpiration and root decay provoked by progressive leaf excision or plant decapitation. From a methodological perspective, the present observations were expected to strengthen the link between root physiology and dielectric properties, and thus authenticate the measurement as a diagnostic tool for the actual root uptake rate. It was thus hoped to provide a smart non-destructive technique to capture the rapid response of root function to altered environmental conditions.

## Materials and methods

### Plant material and cultivation

Two pot experiments were performed on C4 maize (*Zea mays* L., cv. Mv Tarján), and C3 cucumber (*Cucumis sativus* L., cv. Perez-F1; indeterminate growth) and pea (*Pisum sativum* L., cv. Rhine dwarf). The plants were cultivated in 2.9 L plastic pots filled with 1800 g of a 1:1.5 v/v mixture of 0–5 mm rhyolite (Colas Co. Ltd., Tarcal, Hungary) and vermiculite (Pull B.V., Rhenen, The Netherlands). The substrate had a pH_H2O_ of 7.86, cation exchange capacity of 7.88 mmol 100 g^–1^, bulk density of 0.63 g cm^–3^ and 0.33 cm^3^ cm^–3^ water content at field capacity. Its application mostly eliminates the confounding effects derived from the capacitive behavior of complex field soils or organic substrates on root electrical response.

The seeds were germinated on wet paper towels at 22 °C for 3 days, after which the seedlings were planted one per pot to a depth of 2 cm. The plants were cultivated in a growth chamber at 25/17 °C with 16/8 h light/dark cycles (light from 0:00 a.m. to 4:00 p.m.), ~ 600 µmol m^–2^ s^–1^ PAR and 50–70% relative humidity. The substrate was watered daily to 80% of field capacity on a balance (pots were watered from below only from the third week) and fertilized twice a week with 150 mL of Hoagland’s solution. As only one plant could be measured with the instrument on each occasion, a single specimen of a single species was planted each week and grown for 30 days, which was the optimal age for electrical measurement. The phenophase at this time was the 6-leaf stage for maize, the early flowering stage for cucumber (14–15 leaves) and the stem elongation stage for pea (8–9 leaves). In this manner, it was ensured that a 30-day-old plant was available each week for the 7-day electrical measurement procedure.

### Electrical measurement

As a first step, a specialized measurement system was assembled (Fig. 1c). The handheld GW-Instek LCR 916 meter (GoodWill Co. Ltd., Taiwan) was chosen due to its applicability for the real-time monitoring of dielectric variables inside a growth chamber. The impedance response, modelled by a parallel RC circuit, was measured at an AC frequency of 1 kHz with a 1 V terminal voltage. The ground electrode was a stainless steel rod, 15 cm long and 6 mm i.d. (303S31; RS Pro GmbH., Gmünd, Austria), inserted vertically into the substrate to 12 cm depth 5 cm away from the stem base. The plant electrode was clamped 1 cm above the substrate surface through a 4 mm wide, 25 µm thick alumina strip that bent the stem. Although electrode polarization, cable inductance and stray capacitances could not be excluded using the portable LCR 916 device, previous studies [12, 13] convincingly demonstrated that these systematic errors were negligible and did not influence plant dielectric response around the 1 kHz range. In the case of maize, senescencing leaves 1 and 2 were removed to ensure good electric contact. A CRM-2H asymmetric cycler (ETI d.d., Izlake, Slovenia), set to 0.5/5.5 min pulse/pause intervals, was installed between the LCR terminal and the ground electrode. Considering that an AC signal is continuously generated by the LCR meter during the operation time, the cycler served to protect the sample plant against long-term electrical excitement. However, the deterioration of plant life functions is unlikely due to the very small current density formed on the membranes [19]. The LCR instrument was connected to a laptop running LCR900 v.1.201 data logging software.

Twenty-four hours before starting the electrical measurement, the pot was placed in a tray filled with water at a steady depth of 6 mm to adjust the substrate water content (SWC) and keep it constant throughout the measurement period. The water content in the bulk substrate (0–12 cm; the layer of the ground electrode) was 0.26 ± 0.01 cm^3^ cm^–3^, which was checked daily using a HS2 TDR meter attached to a CS659 probe (Campbell Inc., Logan, UT, USA). Nevertheless, this procedure allowed the substrate surface to dry to 0.12 ± 0.01 cm^3^ cm^–3^ at 0–1 cm (checked with an MO750 meter; Extech Co. Ltd., Nashua, NH, USA) to minimize AC leakage [16]. Each electrical measurement started and ended in the middle of the 16-h light period (at 8:00 a.m.) and lasted for 144 h. Ten C_R_ and G_R_ data were collected hourly, synchronizing each reading to a pulse interval of the cycler. As some fluctuations were observed in the collected data, presumably due to variations in temperature and ventilation conditions in the growth chamber, five consecutive data points (covering a 30 min period) were averaged. A G_R_ value was calculated (= C_R_ × D_R_ × ω) from each data pair according to the parallel RC model.

### Diurnal changes (“DC”) experiment

**Fig. 2:**
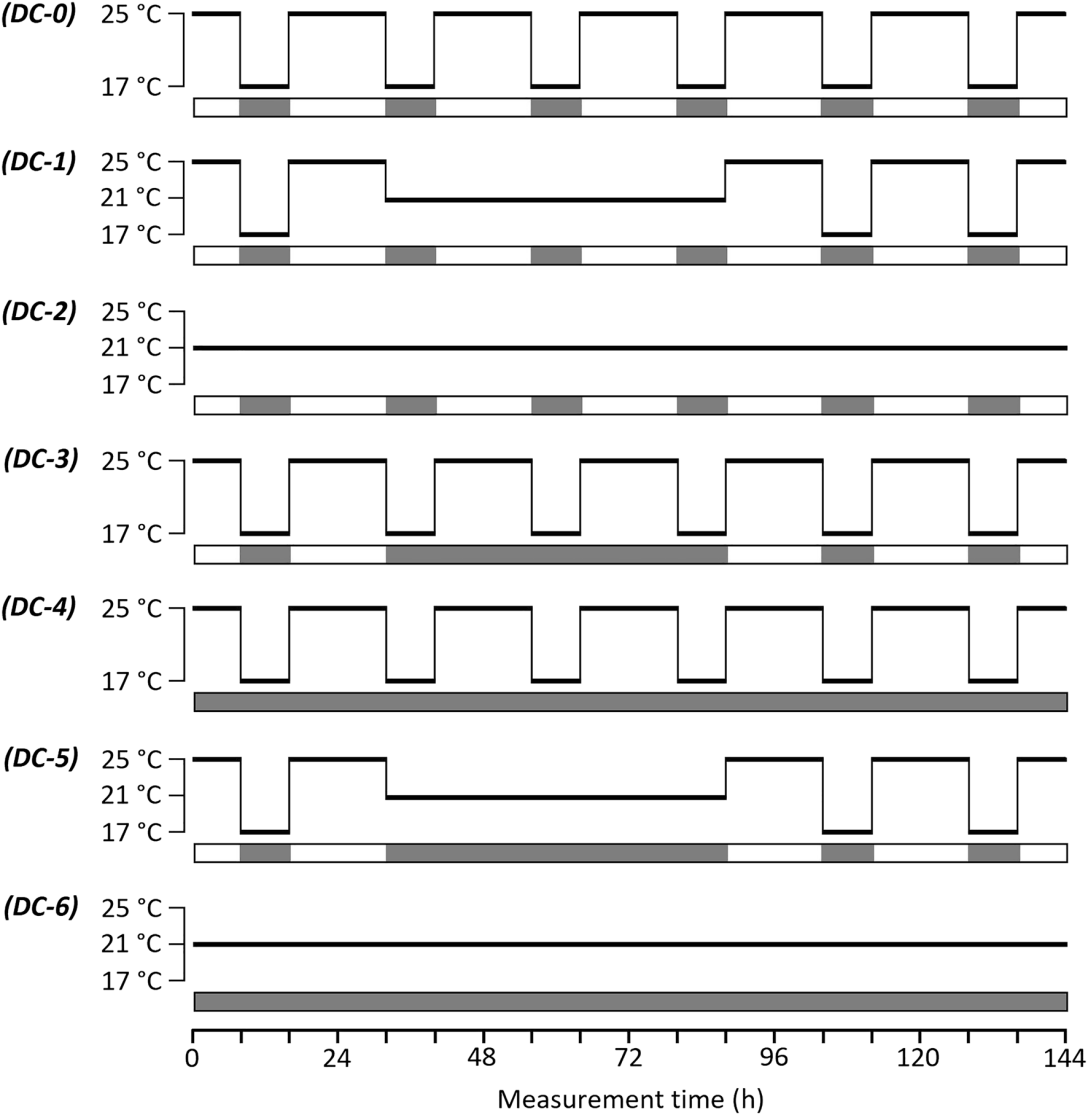
Temperature and light conditions prevailing during the dielectric measurements in the diurnal changes (“DC”) experiment. The gray strips represent the dark periods. See the text for the treatment codes (written in bold in brackets)

Seven plants of each of the three species were cultivated sequentially for the electrical measurements. The first plants (“DC-0”), which served as controls, were measured under normal conditions, *i.e*. 25/17 °C and 16/8 h light/dark cycles for six days (Fig. 2). The following plants were exposed to: constant 21 °C temperature between hours 32–88 with a normal photoperiod (“DC-1”); constant 21 °C between hours 0–144 with a normal photoperiod (“DC-2”); constant dark between hours 32–88 with a normal temperature regime (“DC-3”); constant dark between hours 0–144 with a normal temperature regime (“DC-4”); constant 21 °C and dark between hours 32–88 (“DC-5”); constant 21 °C and dark between hours 0–144 (“DC-6”).

To avoid disturbing (*e.g*. touching) the plants or altering the chamber conditions during electrical monitoring, three additional replicates per species were grown for 30 days to perform shoot physiological investigations. The transpiration rate was characterized by measuring g_s_ using a calibrated SC-1 steady-state diffusion porometer (Decagon Devices Inc., Pullmann, WA, USA). The measurements were taken on leaves 3–6 for maize (numbered from the oldest to the youngest), 4, 6, 8, 10, 12 and 14 for cucumber and 3–8 for pea, in the middle portion of the abaxial (lower) side of the leaves, avoiding margins and main veins. First, mid-light (approximately 8 a.m.) and mid-dark (approximately 8 p.m.) values of g_s_ were assessed at 25 °C and 17 °C, respectively, and then both measurements were repeated the next day at 21 °C.

Stomatal conductance was also determined on the plants subjected to 6-day darkness (“DC-4” and “DC-6”) both before and at the end of the treatment period. In these plants the effect of the long dark period on the photosynthetic apparatus was also evaluated by using two sensitive stress indicators, such as the chlorophyll concentration index (CCI) and the maximum quantum yield of PSII photochemistry, quantified as chlorophyll fluorescence parameter, F_v_/F_m_ [30]. CCI was non-destructively estimated before and after the treatment with a CCM-200^+^ handheld instrument (Opti-Sciences Inc., Hudson, NH, USA), on the adaxial (upper) side of the same leaves used for stomatal investigation. Three CCI readings were averaged for each leaf. F_v_/F_m_ was also determined on these leaves before and after treatment using an OS-30p^+^ fluorometer (Opti-Sciences Inc.). The leaves were first dark-adapted for 20 min using black plastic clamps (9 mm i.d.) and were then exposed to a 1.0 s saturating radiation pulse of 6000 µmol m^–2^ s^–1^ to obtain F_v_/F_m_ values.

### Leaf excision (“LE”) experiment

Six plants of each of the three species were cultivated sequentially and then electrically measured under normal conditions (25/17 °C and 16/8 h light/dark). The first plants (“LE-0”) were untreated controls. The next four (“LE-1”, “LE-2”, “LE-3” and “LE-4”) were subjected to leaf excision, removing a gradually increasing proportion of the total leaf area (TLA) from the subsequent plants with scissors (see Table 2 for details). The last plants (“LE-5”) were decapitated by cutting the stem 1 cm above the plant electrode. The treatments were always performed just after recording the first electrical data (at 0.5 h). The area of excised leaves was determined by scanning them with an LI-3000 instrument (LI-COR Inc., Lincoln, NE, USA) immediately after cutting. The remaining leaves were cut and measured at the end of electrical monitoring, and the ratio of the excised leaf area (rELA) was calculated.

**Table 2:** Specifications for the leaf excision (“LE”) experiment. Leaves are numbered from oldest to youngest

Treatment code	Maize			Cucumber			Pea		
	Excised leaf	TLA (cm^2^)	rELA (%)	excised leaf	TLA (cm^2^)	rELA (%)	excised leaf	TLA (cm^2^)	rELA (%)
LE-0	None	420	0	None	2518	0	None	462	0
LE-1	3	396	9	1–3	2441	15	1–3	520	17
LE-2	3–4	375	41	1–5	2290	48	1–5	545	42
LE-3	3–5	337	79	1–8	2538	81	1–7	481	55
LE-4	3–6	394	94	1–12	2318	92	1–9	505	76
LE-5	Decap	369	100	Decap	2329	100	Decap	471	100

TLA: total leaf area; rELA: ratio of excised leaf area; decap: decapitated plant. See the text for the treatment codes

### Data analysis

The STL seasonal-trend decomposition procedure based on locally estimated scatterplot smoothing (LOESS) was used to extract the additive seasonal part from the time series of C_R_, D_R_ and G_R_ [31]. The percentage of seasonality (PCT), which is the ratio of the variance due to seasonality to the total variance, was calculated. For the sake of clarity, the term “seasonality” was used instead of “cycle” to describe the 24-h fluctuations, since the word “cycle” is used when data exhibit rises and falls that are not of fixed period. Periodogram methods for spectral decomposition were applied in time series analysis [32]. If the data contain strong periodic components, the periodogram values corresponding to those frequencies will be large. The statistical index “s24” was constructed to operationalize this concept: it is a fraction obtained by dividing the values of spectral density attributed to the period of 24 h by the sum of all other values. The 24-h seasonality was rejected if s24 < 2 and was accepted if s24 ≥ 2. It should be noted that the seasonal-trend decomposition extracts a seasonal part, irrespective of whether there is seasonality or not. The scaling of the STL graph is also misleading, always leaving the same part of the seasonality curve. This is why decisions on seasonality were based solely on the s24 index.

To test for monotonicity, short-term fluctuations in the time series had to be ignored. When seasonality was accepted, it was sufficient to check the trend component for monotonicity. When seasonality was rejected, a series of nonlinear kernel smoothing was performed with up to 24-h bandwidth, and monotonicity was only accepted if a monotonic curve was obtained after smoothing. The R^2^ values were reported in this case, but only as supplementary information. The models were essentially nonlinear, so the R^2^ concept of OLS regression should not be adopted unchanged [33]. For the “LE” experiment, the order of the time series was counted at each of the 288 time points, and the counts for the three most frequent orders were listed. To eliminate the plant size effect, C_R_, D_R_ and G_R_ values at each time point were divided by the corresponding initial value (t = 0.5 h), and the resulting relative C_R_, D_R_ and G_R_ values (as a %) were used in this analysis. Statistics were performed using the “stl” function of R 4.0.5 [34], the “periodogram” function of the “descomponer” package, the “npregbw” function of the “np” package and Microsoft Excel.

A two-way comparative assessment was performed to show the influence of illumination and temperature on the root electrical properties. The first categorization of treatments aimed to study the effect of “disturbance” (that is cycles of the factor were replaced by a constant between hours 32–88, and left unchanged elsewhere) as follows: both light/dark and temperature cycles were normal (DC-0); light/dark cycles were normal, but temperature cycles were disturbed (DC-1); light/dark cycles were disturbed, but temperature cycles were normal (DC-3); both light/dark and temperature cycles were disturbed (DC-5). The second categorization was devoted to study the effect of “removal” (that is cycles of the factor were replaced by a constant between hours 0–144) as follows: both light/dark and temperature cycles were normal (DC-0); light/dark cycles were normal, but temperature cycles were removed (DC-2); light/dark cycles were removed, but temperature cycles were normal (DC-4); both light/dark and temperature cycles were removed (DC-6). It should be noted that our experimental design did not allow the estimation of the main effects or interaction effects of factors in the way that is usual for the *e.g*. two-factor ANOVA.

Repeated-measures ANOVA was performed with Tukey’s post-hoc test to study the effect of different temperature and light conditions on the g_s_ of various leaves. The effect of 6-day dark exposition on CCI, F_v_/F_m_ and g_s_ was analyzed using the paired t test. Linear regressions were used to relate g_s_ to both C_R_ and G_R_.

## Results

### Diurnal changes (“DC”) experiment

**Fig. 3:**
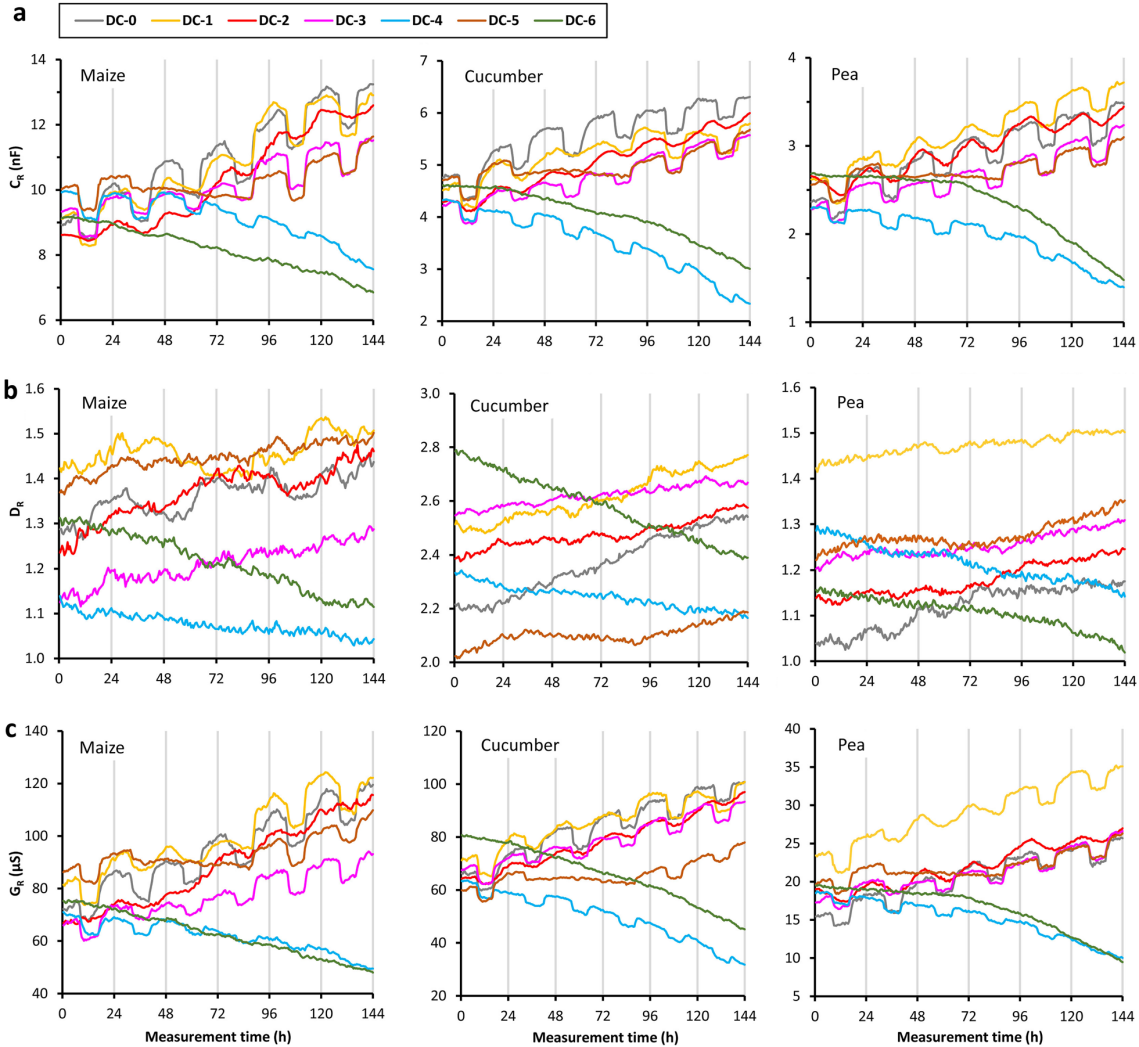
Time series of **a** root electrical capacitance (C_R_), **b** dissipation factor (D_R_) and **c** electrical conductance (G_R_) for maize, cucumber and pea in the diurnal changes (“DC”) experiment. See the text for the treatment codes

The C_R_ value for the control plants (DC-0) showed sharp increases and decreases after the beginning of the light and dark periods, respectively, under the 25/17 °C temperature regime (Fig. 3a), whereas a quasi-sinusoidal diurnal fluctuation with a smaller amplitude was observed for 21/21 °C (DC-1 and DC-2). Compared to the controls, alternating temperature caused fewer rapid changes in C_R_ under extended dark conditions (DC-3 and DC-4). The time series smoothed out under constant 21 °C and in darkness (DC-5 and DC-6).

The statistical results for the C_R_, D_R_ and G_R_ data series are summarized in Table 3 and are available in graphic form for each plant as supplementary materials (Additional files 1, 2, 3, and 4). There was a marked 24-h seasonality in the C_R_ time series in many cases (s24: 2.12–9.19) except for DC-4 maize (s24: 1.88) and for all the plants exposed to prolonged darkness at constant 21 °C (DC-5 and DC-6; s24: 0.03–1.17). PCT ranged from 14.8 to 17.7% for the DC-0 plants (at 25/17 °C), but only reached between 0.9 and 5.5% in the DC-2 treatments (at 21/21 °C). The trend shown by the C_R_ data series was usually increasing, but was decreasing for the plants exposed to 6-day darkness (DC-4 and DC-6).

**Table 3:** Statistical results for the time series of root electrical capacitance (C_R_), dissipation factor (D_R_) and electrical conductance (G_R_) for the three species in the diurnal changes (“DC”) and leaf excision (“LE”) experiments. See the text for the treatment codes

Para-meter	Treatment code	Maize			Cucumber			Pea		
		s24	PCT	Tr	s24	PCT	Tr	s24	PCT	Tr
C_R_	DC-0	**4.69**	14.8	I	**4.60**	17.7	I	**3.04**	17.5	I
	DC-1	**3.63**	9.3	I	**2.93**	16.8	N	**3.04**	8.4	I
	DC-2	**5.05**	0.9	I	**9.19**	1.9	I	**7.23**	5.5	I
	DC-3	**2.83**	21.0	I	**3.41**	11.6	I	**3.04**	17.7	I
	DC-4	1.88	9.1	D	**2.60**	3.3	D	**2.12**	4.3	D
	DC-5	1.17	21.0	N	1.06	13.8	I	1.10	18.7	I
	DC-6	0.72	0.3	D	0.10	0.1	D	0.03	0.1	D
	LE-0	**5.86**	28.8	I	**4.67**	22.9	I	**3.09**	14.3	I
	LE-1	**7.08**	24.5	I	**4.39**	12.5	I	**4.32**	14.6	I
	LE-2	**5.06**	28.3	I	**5.05**	16.8	I	**3.86**	67.4	N
	LE-3	**4.11**	31.0	N	**4.82**	39.8	N	**3.81**	38.2	N
	LE-4	**3.02**	29.6	N	**3.72**	59.8	N	**2.07**	52.3	N
	LE-5	0.28	1.3	N	0.19	1.9	N	0.11	2.0	N
D_R_	DC-0	0.44	5.3	I	0.14	0.1	I	0.64	1.6	I
	DC-1	0.51	6.4	N	0.47	0.6	I	0.08	0.9	I
	DC-2	0.03	0.8	I	0.37	1.3	I	0.26	0.7	I
	DC-3	0.35	2.3	I	0.81	1.9	I	0.03	0.4	I
	DC-4	0.21	2.3	D	0.06	0.7	D	0.02	0.3	D
	DC-5	0.60	2.8	I	0.02	0.7	I	0.02	0.9	I
	DC-6	0.10	0.4	D	0.01	0.1	D	0.02	0.4	D
	LE-0	0.90	2.6	I	0.39	0.4	I	0.54	0.8	I
	LE-1	0.49	1.2	I	1.19	1.6	I	1.59	3.8	N
	LE-2	0.03	0.2	N	1.11	2.5	I	1.99	26.8	N
	LE-3	0.07	0.4	I	0.52	1.5	I	0.35	3.7	N
	LE-4	0.16	0.2	I	0.92	6.7	I	0.19	0.6	D
	LE-5	0.01	0.1	I	0.69	0.3	I	0.04	0.0	N
G_R_	DC-0	**4.82**	13.1	I	**4.83**	8.5	I	**3.23**	11.7	I
	DC-1	**3.30**	9.9	I	**3.38**	10.3	I	**3.53**	7.1	I
	DC-2	1.90	0.5	I	**8.39**	1.5	I	**5.81**	3.7	I
	DC-3	**2.98**	13.1	I	**3.49**	9.6	I	**3.03**	12.5	I
	DC-4	1.72	7.4	D	**2.42**	2.8	D	1.79	3.0	D
	DC-5	1.31	14.6	I	1.12	8.5	I	1.04	10.2	I
	DC-6	0.51	0.3	D	0.07	0.1	D	0.02	0.1	D
	LE-0	**6.68**	20.9	I	**4.96**	12.8	I	**3.46**	8.5	I
	LE-1	**8.22**	16.5	I	**4.90**	9.5	I	**5.68**	11.7	I
	LE-2	**4.49**	15.1	I	**5.56**	13.9	I	**5.08**	71.4	N
	LE-3	**2.86**	16.5	N	**5.35**	27.4	N	**4.21**	44.7	N
	LE-4	**2.29**	7.4	N	**4.23**	50.5	N	1.89	22.2	D
	LE-5	0.37	0.5	I	0.37	1.9	N	0.11	0.3	N

24- h seasonality was accepted (s24 ≥ 2; written in bold)

There was no significant 24-h seasonality in the D_R_ series (s24: 0.01–0.81; Fig. 3b). It exhibited an increasing trend for several plants, including the controls, but a decreasing trend when the 6-day dark treatment was applied (DC-4 and DC-6).

The temporal pattern of G_R_ was evidently quite similar to that of C_R_ in terms of both seasonality and trend owing to the relatively steady D_R_ value (Fig. 3c). The unaltered temperature coupled with dark treatment (DC-5 and DC-6) eliminated the seasonality from the G_R_ data series. A strongly decreasing trend in G_R_ was exhibited by the plants kept in darkness for 6 days (DC-4 and DC-6) due to a reduction in both the C_R_ and D_R_ components over time. The two-way comparative assessment indicated that “disturbance” and “removal” effects on C_R_ and G_R_ were appreciable only when they were concurrently applied to both illumination and temperature.

**Fig. 4:**
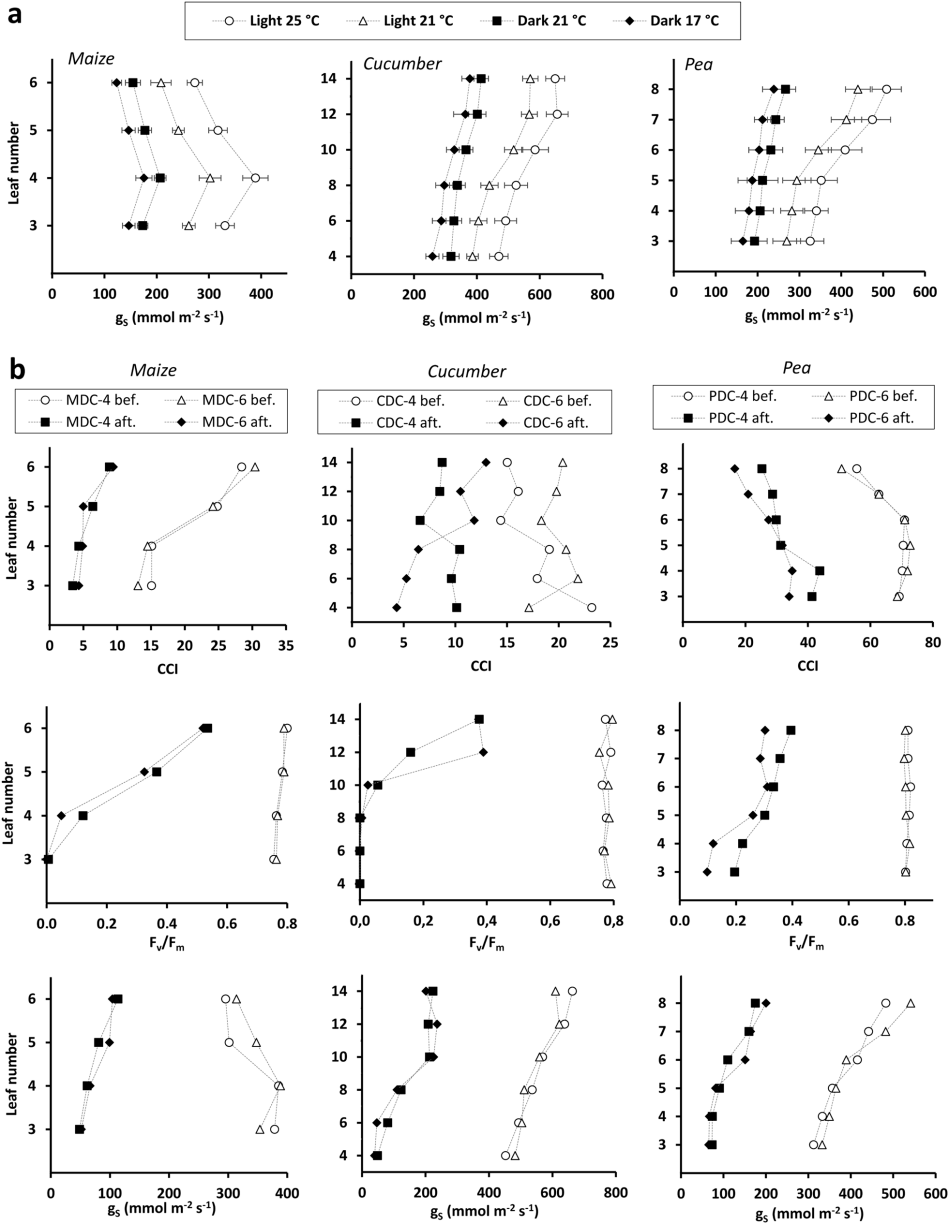
**a** Mid-light and mid-dark stomatal conductance (g_s_) measured on various leaves in maize, cucumber and pea plants (n = 3) at different air temperatures. Leaves are numbered from oldest to youngest. **b** Chlorophyll concentration index (CCI), chlorophyll fluorescence parameter (F_v_/F_m_) and stomatal conductance (g_s_) measured on various leaves in maize, cucumber and pea plants before and after a 6-day dark period in the diurnal changes (“DC”) experiment. See the text for the treatment codes

The g_s_ value measured on different leaves proved to be smaller at lower temperature and in darkness (Fig. 4a). Repeated-measures ANOVA showed that temperature and illumination had a significant (*p* < 0.01) effect on g_s_, except for the mid-dark g_s_ value at 17 °C versus 21 °C for pea (*p* > 0.05). Severe leaf chlorosis and wilting were observed after 6-day darkness (DC-4 and DC-6) for each species. This treatment substantially reduced the CCI, F_v_/F_m_ and g_s_ for each leaf (Fig. 4b). The paired t tests indicated a significant dark effect for all the physiological parameters and species (*p* < 0.001 in most cases). F_v_/F_m_ and g_s_ drastically decreased for maize and cucumber in the older leaves, which were practically dead. Significant linear correlations were found between C_R_ and g_s_ (R^2^: 0.665–0.804; *p* < 0.05; n = 6) and between G_R_ and g_s_ (R^2^: 0.698–0.774; *p* < 0.05; n = 6) for plants subjected to the long dark treatment (Additional file 5). Note that the correlations calculated from 6 data pairs are only indicative, and more reliable statistics can be provided by evaluating further repeated measurements.

### Leaf excision (“LE”) experiment

**Fig. 5:**
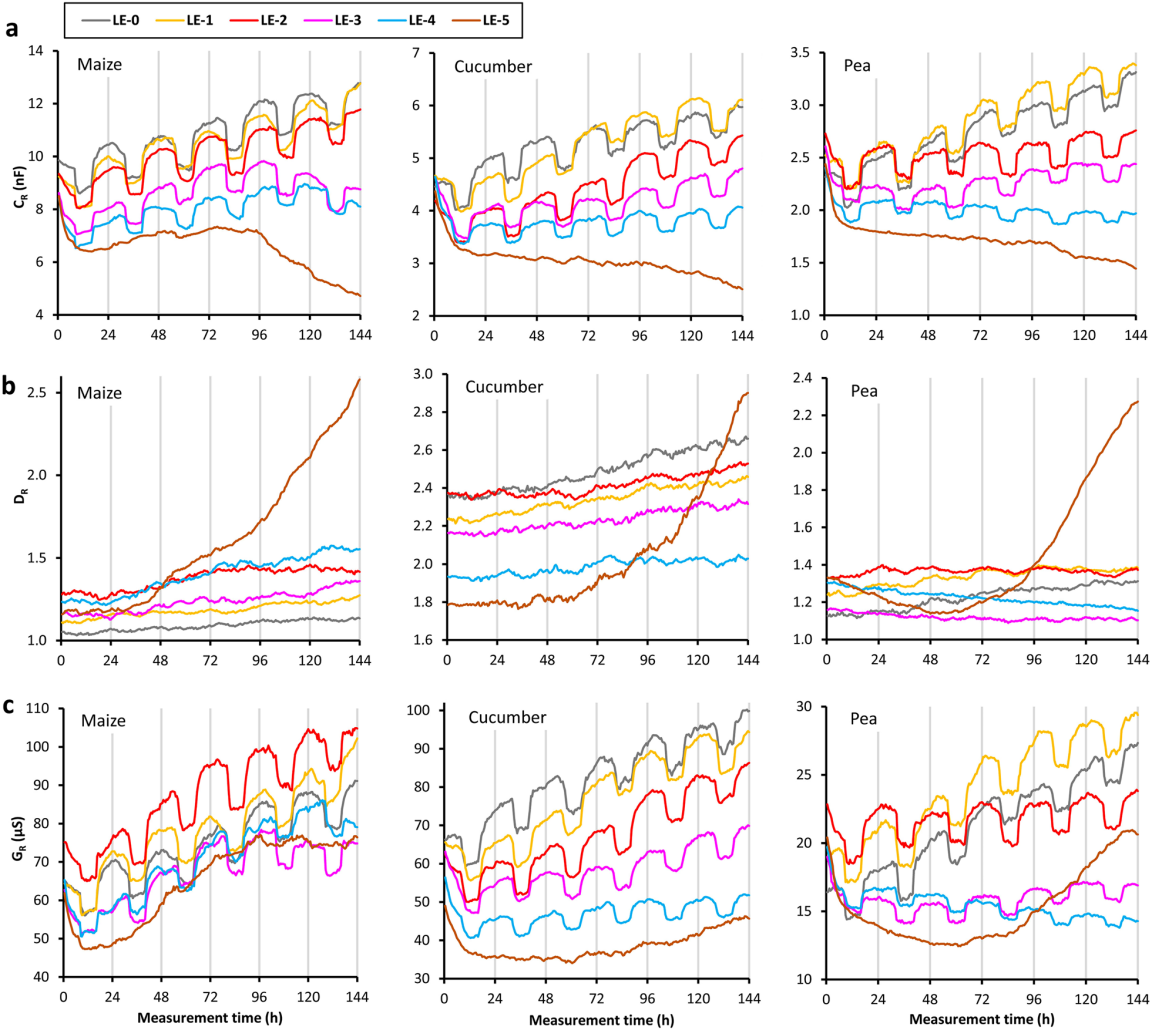
Time series of **a** root electrical capacitance (C_R_), **b** dissipation factor (D_R_) and (**c**) electrical conductance (G_R_) for maize, cucumber and pea in the leaf excision (“LE”) experiment. See the text for the treatment codes

The periodogram method revealed a significant 24-h seasonality in the C_R_ time series for the control and the excised plants (from LE-0 to LE-5), with s24 between 2.07 and 7.08 (Fig. 5a). The degree of seasonality tended to decrease after severe defoliation and ceased after plant decapitation (s24: 0.11–0.28). Leaf excision reduced C_R_ to the greatest extent during the first few hours of measurement, but continued to exert a negative effect for the rest of the time. Therefore, the increasing trend in C_R_ became non-monotonic. As the increasing trend disappeared from the C_R_ data series earlier than seasonality after progressive leaf excision, PCT varied over a wide range from 12.5 to 67.4%. C_R_ decreased rapidly immediately after decapitation, after which it continued to decline moderately. However, in the case of maize, C_R_ increased slightly until approximately t = 96 h, and then started to decline again. A descending order in the relative C_R_ values in relation to increasing rELA and decapitation was observed at the majority of the 288 time points (see Additional file 4 for details).

No seasonality was found in the D_R_ measurement series (s24: 0.01–1.99), but small local maxima in D_R_ were exhibited by some plants (*e.g.* LE-2 pea) during the light hours (Fig. 5b). In general, the trend in the data series was increasing, but was non-monotonic for some pea plants. Furthermore, a drastic increase in D_R_ was observed for the decapitated (LE-5) plants from the second or third day of measurement. The degree of leaf excision did not affect the order of relative D_R_.

The leaf-excised plants exhibited a significant 24-h seasonality in G_R_ (s24: 2.29–8.22), except for the LE-4 pea plant (s24: 1.89) and the decapitated plants (s24: 0.11–0.37; Fig. 5c). As observed for C_R_, the increasing trend in the G_R_ series became non-monotonic after progressive leaf excision. In the decapitated (LE-5) plants, G_R_ increased in the second half of the measurement period (or earlier for maize) due to the marked rise in D_R_, and finally exceeded the values of severely excised plants (especially pea). This finding was confirmed by the order analysis of the relative G_R_ data.

### Relationship between the capacitance, conductance and transpiration losses

**Fig. 6:**
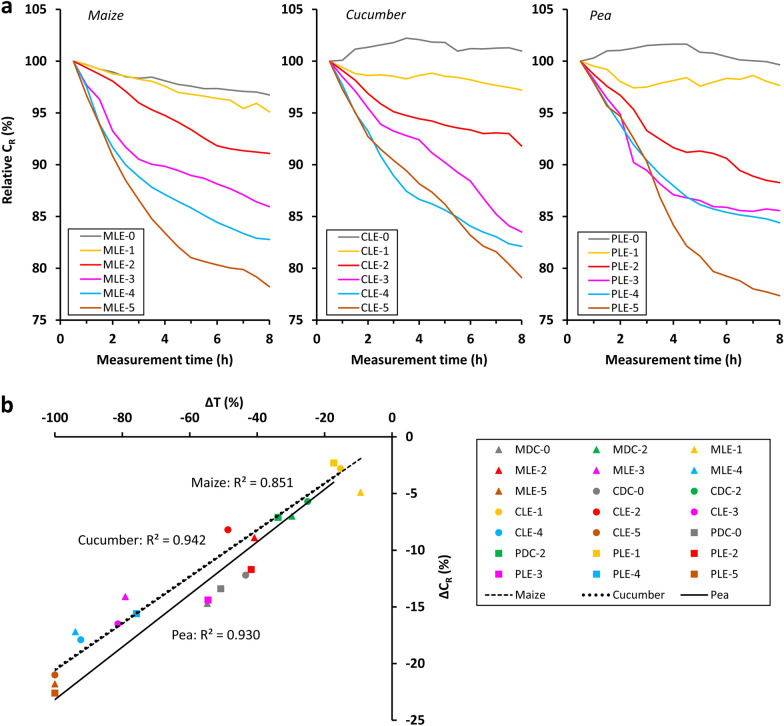
**a** Changes in relative root electrical capacitance (C_R_) during the first eight measurement hours for maize, cucumber and pea in the leaf excision (“LE”) experiment. Relative C_R_ is the ratio of measured C_R_ to the corresponding initial value (t = 0.5 h). **b** Linear relationships between capacitance loss (ΔC_R_) and transpiration loss (ΔT) for the three species. Regressions were significant at the p < 0.01 level. See the text for details

**Fig. 7:**
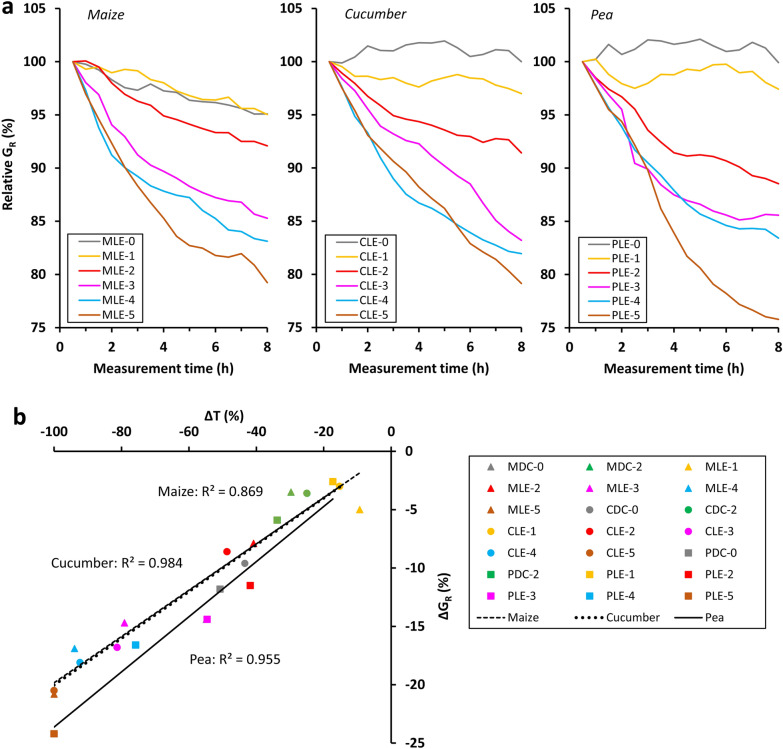
**a** Changes in relative root electrical conductance (G_R_) during the first eight measurement hours for maize, cucumber and pea in the leaf excision (“LE”) experiment. Relative G_R_ is the ratio of measured G_R_ to the corresponding initial value (t = 0.5 h). **b** Linear relationships between electrical conductance loss (ΔG_R_) and transpiration loss (ΔT) for the three species. Regressions were significant at the p < 0.01 level. See the text for details

The measurement series showed a sudden reduction in C_R_ and G_R_ after leaf excision and decapitation, and the rate of decrease seemed to be proportional to the degree of leaf area loss (Figs. 6a, 7a). A ΔC_R_ and ΔG_R_ (%) value was calculated for each excised plant (from LE-0 to LE-5) by deducting the relative C_R_ and G_R_ (%) obtained at the end of the first light period at t = 8 h (to exclude the dark effect) from the initial 100% C_R_ and G_R_, respectively. The transpiration loss, ΔT (%), was also calculated for these plants, which was equal to rELA and was 100% for the decapitated plants (Table 2). The relationships between ΔC_R_ or ΔG_R_ and ΔT were evaluated by regression analysis for each species. DC-0 and DC-2 plants were also included in the investigation. For these plants, ΔC_R_ and ΔG_R_ was the percentage difference between the 6-day means of mid-light and mid-dark C_R_ and G_R_, respectively (Fig. 3a, c), and ΔT was the difference between the mid-light and mid-dark g_s_ values (at 25/17 °C for DC-0 and 21/21 °C for DC-2), obtained by averaging the values measured for the various leaves (Fig. 4a).

Strong linear correlations were found between ΔC_R_ and ΔT (R^2^: 0.851–0.942; *p* < 0.01; n = 7) and between ΔG_R_ and ΔT (R^2^: 0.869–0.984; *p* < 0.01; n = 7) for each species when the regression lines were forced through the origin (Figs. 6b, 7b). The F tests showed that the slope of ΔC_R–_ΔT regression line for pea was marginally significantly (*p* = 0.08) different from those of the other two species, whereas the same comparison proved to be highly significant (*p* < 0.01) for the ΔG_R_–ΔT regression line.

## Discussion

### Trends and light/dark changes in root dielectric properties

In the “DC” experiment, the increasing trend in the C_R_ and G_R_ time series indicated an increase in the active (polarizable) root surface area of the developing plants during the measurement period. The highest rate of change was found for maize, which exhibited intensive vegetative growth at this time. The seasonal patterns in C_R_ and G_R_ were associated with the light/dark and temperature conditions applied. It should be noted, first, that membrane capacitors show a slight reduction in capacitance with rising temperature due to decreasing *ε*_r_* [7] and, second, that although electrical conduction in plants is linked to electrolyte movement [8] and thus increases with temperature, the 24-h seasonality in G_R_ was significant under constant light/dark temperature as well. Therefore, the seasonal changes in dielectric response observed in the present study cannot be explained merely by general physical rules but rather by the temporal pattern of integral root absorption activity.

As only 1–2% of the water taken up by the plant is involved in metabolic processes [35], the actual canopy transpiration is closely related to the water extraction and conductance of the entire root zone [36]. Water flows in the root cylinder through spatially distributed and temporally variable composite, *i.e*. apoplastic and cell-to-cell (including symplastic and transmembrane) pathways, according to physical laws and intrinsic or responsive regulatory mechanisms [37]. The diurnal cycle of water transport within plant organs is controlled by an endogenous circadian oscillator that drives rhythmic gene expressions, causing systematic variations in root membrane permeability (and in turn L_R_), photosynthetic rate, chlorophyll fluorescence and stomatal (leaf hydraulic) conductance [28, 38]. Furthermore, diurnal patterns are strongly modified by prevailing environmental conditions, such as radiation (the primary driver of photosynthesis), air temperature, humidity and ventilation.

Unlike naturally growing plants, which exhibit quasi-sinusoidal diurnal patterns [39], the plants in this experiment showed rapid light/dark changes in C_R_ and G_R_ in the 25/17 °C temperature treatment. This was likely due to the instant shift between total illumination and complete darkness and the short transition in the temperature conditions (only 20–22 and 30–35 min from 25 to 17 °C and vice versa, respectively) in the growth chamber. The sudden changes in C_R_ and G_R_ were eliminated when the light/dark temperature remained constant at 21 °C between 32–88 and 0–144 h, leading to wavy curves during these periods. Under a normal (16/8 h) photoperiod the PCT values for the C_R_ and G_R_ data series were substantially higher at 25/17 °C (8.5–17.7) than at 21/21 °C (0.5–5.5). Correspondingly, the difference between light/25 °C and dark/17 °C canopy transpiration (based upon the g_s_ measurements) was much higher than in the case of light/21 °C and dark/21 °C. The fluctuation in C_R_ and G_R_ ceased under constant darkness at 21 °C, eliminating the seasonality from the time series. Although the circadian rhythm in water transport is maintained for several days under continuous darkness by the aquaporin-based regulation of the symplastic solute (and in turn AC) pathway [39], the two-terminal dielectric measurement seemed to be unable to reveal the cycles in the absence of alternating external conditions.

### Effect of long-term dark

In the present study, the strong adverse effect of the 6-day dark period was evident as chlorotic, wilted or dried leaves and as drastically reduced values of CCI, F_v_/F_m_ and g_s_. The typical consequences of light deprivation include chlorophyll degradation, impaired photosynthetic efficiency, stomatal closure, and subsequent carbon starvation, resulting in reduced shoot and root growth [40, 41]. Impeded root development leads to a higher ratio of older, more suberized root regions to younger segments responsible for water uptake, reducing the integrated absorptive surface area and physiological activity [42]. The deposition of apoplastic barriers in aging roots decreases the radial conductance to water and AC [37]. These effects were clearly shown by the decrease in C_R_ and G_R_ over time. The stress-induced stomatal closure, indicated by markedly reduced g_s_ in the present case, could also have contributed to the decline in root activity [43], as was confirmed statistically by the significant linear regressions between C_R_ and g_s_, and between G_R_ and g_s_.

Alternating temperature caused seasonality in the C_R_ and G_R_ data series during the 6-day dark period, likely due to fluctuating whole-plant transpiration and water uptake rates. The cycle amplitude was smaller than that under a normal photoperiod, and decreased further with ongoing dark-induced senescence, so it was statistically rejected in some cases (s24: 1.72–2.60). These findings are consistent with a previous report showing that resource deprivation decreased the cyclicity of L_R_ [29]. Nevertheless, the decreasing trend in D_R_ for plants exposed to 6-day darkness seems to contradict previous studies that detected enhanced D_R_ in response to stress [17, 18], as was also observed here for the decapitated plants. One hypothetical reason is that dark-induced stomatal closure and leaf senescence restrict apoplastic water movement, chiefly driven by transpiration pull, rather than cell-to-cell transport, which primarily depends on the root pressure generated by osmotic (ion pump) forces [44]. The increased dominance of transmembrane (more capacitive) against apoplastic (electrically more resistive) pathways could result in higher charge-storage (polarization) efficiency, reflected by reduced conductive energy loss (D_R_).

### Leaf excision and decapitation

The relationship of C_R_ and G_R_ to the momentary uptake activity of the root system was further verified by the “LE” experiment. Increasing levels of defoliation caused a gradual decrease in C_R_ and G_R_ for each species, as determined by the order analysis of the measurement series. The likely reason is that leaf removal reduced the total transpirational surface area and, in turn, the whole-plant water uptake rate. The weakening seasonality (s24) in the C_R_ and G_R_ time series along with increasing rELA was thought to be due to the removal of the most photosynthetically active young leaves. Similarly to the effect of dark-induced leaf senescence, severe defoliation levels also cause a limited supply and reallocation of photo-assimilates, resulting in restricted root growth [45, 46]. This is why the increasing trend in the C_R_ and G_R_ measurement series became non-monotonic or even decreasing. Notably, both C_R_ and G_R_ rapidly decreased after leaf excision, and the changes in C_R_ and G_R_ over the remaining part of the first light period (0–8 h) relative to the initial values (ΔC_R_ and ΔG_R_) were linearly correlated with the percentage decrease in canopy transpiration (ΔT, including rELA). This was attributed to the reduced transpiring leaf surface area and the proportionally lower root water uptake and conductivity [44]. Water absorption from the soil and transport into the xylem (root pressure) may continue for many hours after cutting off the shoot [39], as was shown by the exuding stump surface following decapitation for each species. L_R_ can be considerably altered in the space of a few minutes after the disappearance of xylem tension due to the absence of transpiration [37]. It was also shown that shoot excision caused substantial changes in root metabolism and transport processes within 2–6 h of stress [47], influencing potentially the dielectric response. Furthermore, shoot injuries *i.e.* leaf cutting and, in particular, decapitation can directly induce both hyper- and depolarization electrical signals, which could confound root impedance up for an hour after treatment [21].

D_R_ began to increase sharply several days after plant decapitation, indicating membrane disorganization and enhanced water diffusion (higher conductive energy loss), in association with increased aquaporin activity due to the accumulation of reactive oxygen species (ROS) [48]. Serious shoot damage was also reported to initiate programmed cell death through signal-induced excessive ROS production [20]. Such changes in the complex impedance response derived from root tissue modifications have usually been demonstrated in plants subjected to cadmium, alkalinity, drought, or anoxia stress [18, 25, 49–51]. Structural changes in the root cortex, including the reduced volume of living cells and increased wall density, and in turn enhanced apoplastic current flow were suspected to be responsible for smaller *Φ* (higher D_R_) in aging maize roots [52]. Extensive root death was observed within 4–5 days following the excision of clover, maize and tomato shoots due to the rapid depletion of soluble carbohydrate reserves [53]. Root decay was clearly shown by the gradually decreasing C_R_ over time in the present case. The enhanced water flux through the deteriorated membrane structures provoked a transient increase in C_R_, followed by a further decrease later because of the ongoing plant senescence. Interestingly, C_R_ increased until the fourth day of measurement in decapitated maize, while rapid resprouting was observed, but the new shoot tip had died by the end of the dielectric monitoring. Monocots tolerant to severe defoliation are reported to exhibit increased root metabolic activity and reserve remobilization to satisfy the resource demands of the regrowing shoot [45].

### Methodological implications

The above results from the two experiments basically concur with Dalton’s [6] model, implying that roots in the substrate behave as lossy capacitors in the electrical circuit, and that C_R_ and G_R_ are valid indicators of root physiological activity. However, further studies are necessary to clarify the role of changing capacitor plate (absorption zone) area (A) and changing permittivity (*ε*_r_) in diurnal fluctuations. The light/dark changes in dielectric variables in plants measured under normal and modified temperature and illumination conditions, and the reduction in C_R_ and G_R_ after defoliation strongly suggest direct links between the impedance components and the momentary water uptake rate of the whole root system. This finding was further supported by the good correlations between C_R_ or G_R_ and g_s_ in the case of dark treatments (R^2^ from 0.665 to 0.804), and also by the significant linear relationships found between ΔC_R_ or ΔG_R_ and ΔT (R^2^ from 0.851 to 0.984), when all the leaf-excised and some intact plants (DC-0 and DC-2) were included in the data analysis. It is true that ΔT was only an estimate based on measuring g_s_, without considering the different surface areas of individual leaves, the stem transpiration, and the potential further transpiration loss after the end of the first light period. Nevertheless, the present results verified that the dielectric properties generated depend on the extension and functionality of the root system. The established regressions imply a 20–25% contribution of the actual water uptake activity to the C_R_ and G_R_ values detected. However, the F test results suggest the effect of species on the regression slopes. Although the C3 and C4 plants show inherent differences in morpho-anatomical and physiological features and response to abiotic stresses [30, 39], their dielectric characteristics seemed basically similar.

The present observations on the dielectric response contradict a previous statement that roots below the substrate surface play a negligible role in C_R_, which originates only from stem-base polarization [9]. However, the results partly agree with the assumptions of Dietrich et al.’s [9] alternative model, as shoot tissues below the plant electrode act as series-connected capacitors, and the subcircuit comprising the stem base and surface roots could strongly influence total capacitance. In contrast, it was reported previously that the preferable current paths are jointly determined by the radial and longitudinal conductivity of the root and the resistance of the surrounding substrate [15, 16]. Our findings confirm these studies, showing that—under relatively dry substrate surface conditions—AC flow is at least partially distributed throughout the root system, which in turn can be directly detected. We cannot comment further regarding on what degree the diurnal pattern and stress-induced changes in C_R_ and G_R_ were due to the altered uptake rate of distal fine roots, as their contribution to the electrical circuit seemed to be uncertain [10, 13]. Nevertheless, a substantial current leakage from the substrate surface, and the dominance of stem-base polarization in the dynamically changing root dielectric response obtained is implausible. A previous root excision experiment with potted maize revealed that the root system within the substrate was responsible for approximately two-thirds of the magnitude of C_R_ [14]. Moreover, the current fine-time monitoring study provided evidence that the dielectric response measured was influenced not only by the geometric size but also by the momentary uptake intensity of the root system.

## Conclusions and future prospects

In this exploratory research, a customized, innovative measurement system was tested for the low-frequency, two-terminal dielectric monitoring of intact root–substrate systems at fine-time resolution in a laboratory environment. The dynamic measurement of the impedance response proved to be an efficient diagnostic tool to follow the changes in integrated root absorption activity, including the diurnal pattern, as well as to track the effect of altered temperature and light/dark conditions, and the reduced canopy transpiration provoked by various levels of defoliation. Since this novel technique requires neither multi-frequency (spectral) instrument, nor multi-electrode (tomographic) arrangement, it can be completed inside the growth chamber using a cheap handheld LCR device. The real-time data logging allows for capturing and analyzing quick changes in plant uptake activity without damaging plant structures or affecting life functions. Diurnal changes could be directly detected by measuring the root dielectric characteristics, in contrast with previous approaches based on following SWC pattern around the roots to obtain soil water acquisition. It was demonstrated that—in compliance with the basic assumption of measuring roots—the dielectric properties detected in situ mainly originated from root tissue polarization, and were influenced by actual physiological activity. However, confirmatory research data from more plant replicates are needed to support some present findings. Standardization of electrode type and positioning is relatively simple, but maintenance of stable and appropriate SWC requires a careful planning. The electrical approach for intact roots is still in the experimental stage, without a full understanding of its basic physics. It is a challenging task to directly link impedance parameters to whole-plant transpiration and integrated root water uptake and conductance, *e.g*. using the dielectric technique concurrently with the plant chamber or sap flow method in the same plant. This may open the door to resolve scientific debates on the preferred electrical pathways and method efficiency, and to establish improved conceptual models for the underlying biophysical mechanisms. Moreover, the results could contribute to the universal acceptance of the dielectric approach for evaluating root physiological status and environmental response in situ.

### Supplementary Information

**Additional file 1: **Periodograms for the time series of root electrical capacitance (C_R_), dissipation factor (D_R_) and electrical conductance (G_R_).

**Additional file 2: **STL seasonal-trend decomposition for the time series of root electrical capacitance (C_R_), dissipation factor (D_R_) and electrical conductance (G_R_).

**Additional file 3: **Monotonicity tests by nonlinear kernel smoothing with up to 24-h bandwidth for the time series of root electrical capacitance (C_R_), dissipation factor (D_R_) and electrical conductance (G_R_).

**Additional file 4: **Order analysis for the time series of root electrical capacitance (C_R_), dissipation factor (D_R_) and electrical conductance (G_R_).

**Additional file 5: **Linear regressions between root electrical capacitance (C_R_) and stomatal conductance (g_s_) and between root electrical conductance (G_R_) and g_s_ for plants subjected to 6-day dark period.

## Data Availability

All data generated or analyzed during this study are included in this published article and its Additional information files.
