# Supplementary figures and images for: Monitoring of plant water uptake by measuring root dielectric properties on a fine timescale: diurnal changes and response to leaf excision (part 3 of 4)
Source: Plant Methods. 2024 Jan 9;20:5. doi: 10.1186/s13007-023-01133-8 (PMC10775601; doi:10.1186/s13007-023-01133-8)

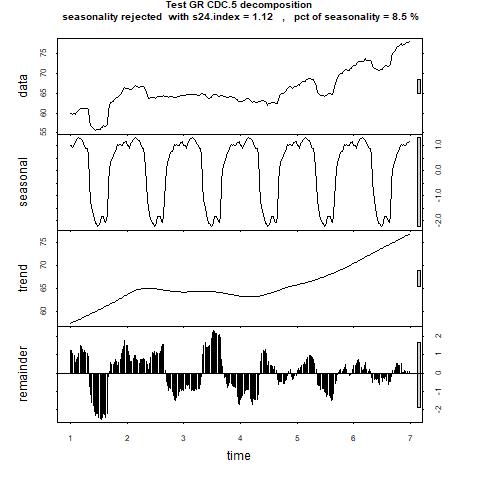

Supplement: Supplementary file 2 — Additional file 2: STL seasonal-trend decomposition for the time series of root electrical capacitance (CR), dissipation factor (DR) and electrical conductance (GR). [file 13007_2023_1133_MOESM2_ESM.zip › GR_CDC5.jpg]

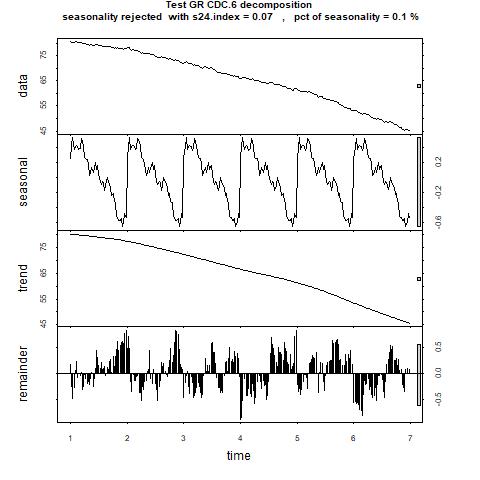

Supplement: Supplementary file 2 — Additional file 2: STL seasonal-trend decomposition for the time series of root electrical capacitance (CR), dissipation factor (DR) and electrical conductance (GR). [file 13007_2023_1133_MOESM2_ESM.zip › GR_CDC6.jpg]

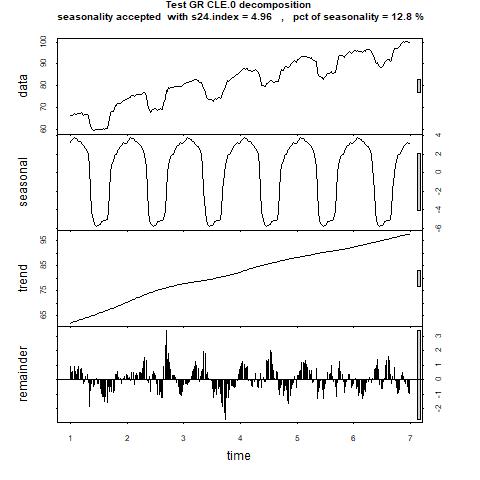

Supplement: Supplementary file 2 — Additional file 2: STL seasonal-trend decomposition for the time series of root electrical capacitance (CR), dissipation factor (DR) and electrical conductance (GR). [file 13007_2023_1133_MOESM2_ESM.zip › GR_CLE0.jpg]

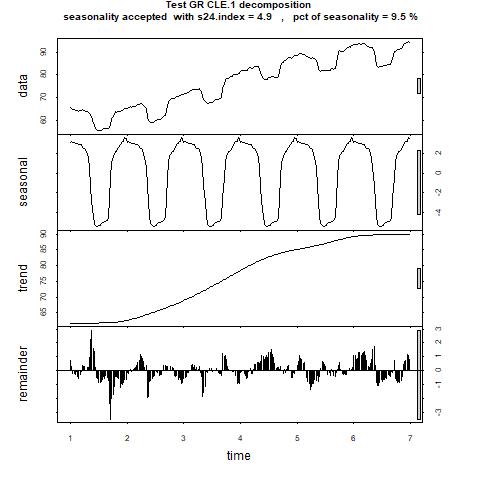

Supplement: Supplementary file 2 — Additional file 2: STL seasonal-trend decomposition for the time series of root electrical capacitance (CR), dissipation factor (DR) and electrical conductance (GR). [file 13007_2023_1133_MOESM2_ESM.zip › GR_CLE1.jpg]

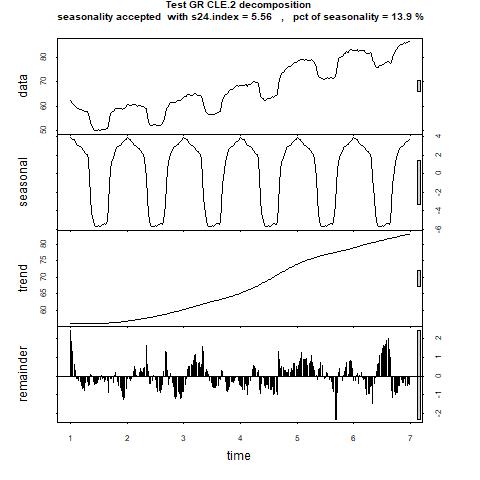

Supplement: Supplementary file 2 — Additional file 2: STL seasonal-trend decomposition for the time series of root electrical capacitance (CR), dissipation factor (DR) and electrical conductance (GR). [file 13007_2023_1133_MOESM2_ESM.zip › GR_CLE2.jpg]

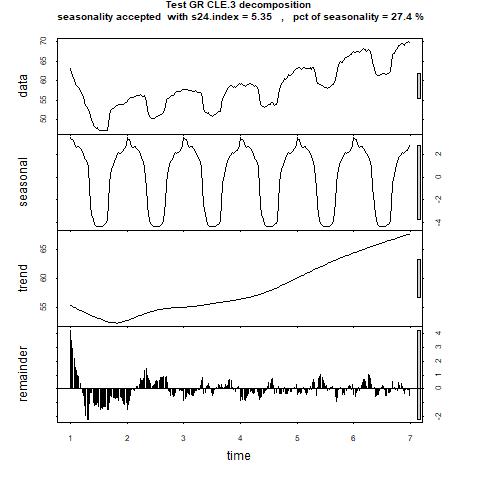

Supplement: Supplementary file 2 — Additional file 2: STL seasonal-trend decomposition for the time series of root electrical capacitance (CR), dissipation factor (DR) and electrical conductance (GR). [file 13007_2023_1133_MOESM2_ESM.zip › GR_CLE3.jpg]

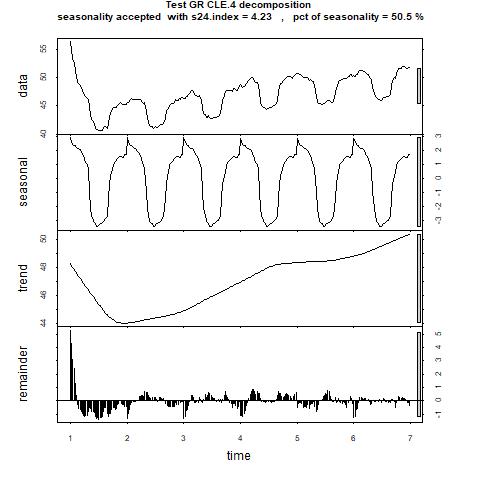

Supplement: Supplementary file 2 — Additional file 2: STL seasonal-trend decomposition for the time series of root electrical capacitance (CR), dissipation factor (DR) and electrical conductance (GR). [file 13007_2023_1133_MOESM2_ESM.zip › GR_CLE4.jpg]

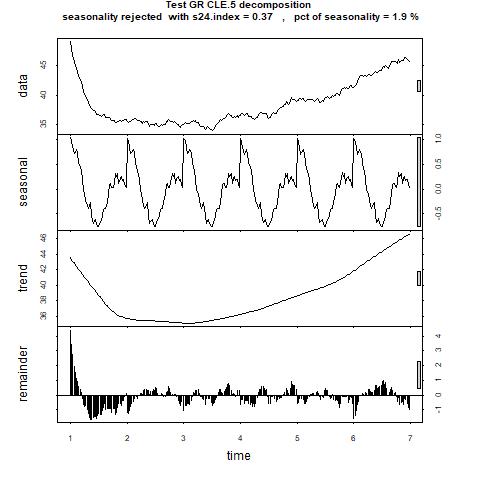

Supplement: Supplementary file 2 — Additional file 2: STL seasonal-trend decomposition for the time series of root electrical capacitance (CR), dissipation factor (DR) and electrical conductance (GR). [file 13007_2023_1133_MOESM2_ESM.zip › GR_CLE5.jpg]

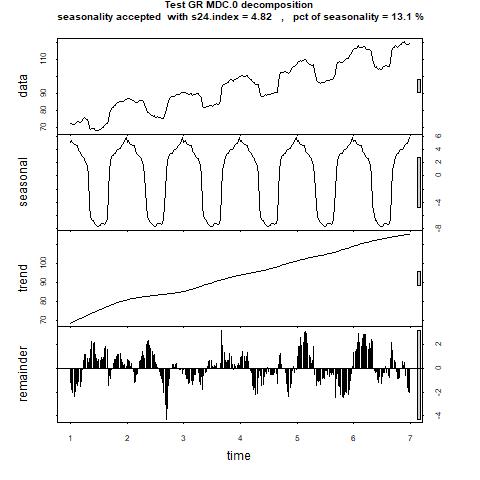

Supplement: Supplementary file 2 — Additional file 2: STL seasonal-trend decomposition for the time series of root electrical capacitance (CR), dissipation factor (DR) and electrical conductance (GR). [file 13007_2023_1133_MOESM2_ESM.zip › GR_MDC0.jpg]

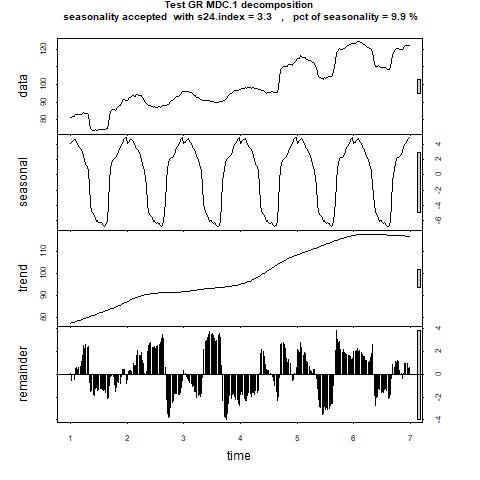

Supplement: Supplementary file 2 — Additional file 2: STL seasonal-trend decomposition for the time series of root electrical capacitance (CR), dissipation factor (DR) and electrical conductance (GR). [file 13007_2023_1133_MOESM2_ESM.zip › GR_MDC1.jpg]

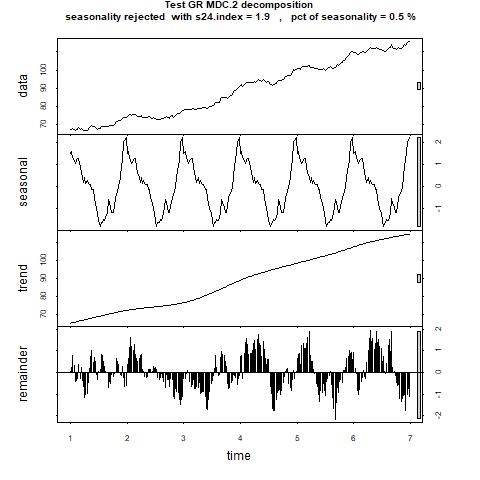

Supplement: Supplementary file 2 — Additional file 2: STL seasonal-trend decomposition for the time series of root electrical capacitance (CR), dissipation factor (DR) and electrical conductance (GR). [file 13007_2023_1133_MOESM2_ESM.zip › GR_MDC2.jpg]

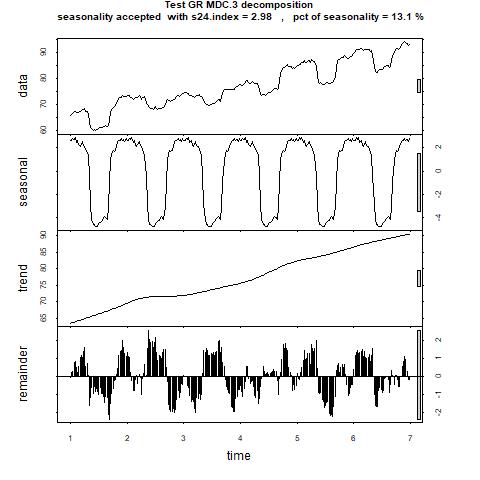

Supplement: Supplementary file 2 — Additional file 2: STL seasonal-trend decomposition for the time series of root electrical capacitance (CR), dissipation factor (DR) and electrical conductance (GR). [file 13007_2023_1133_MOESM2_ESM.zip › GR_MDC3.jpg]

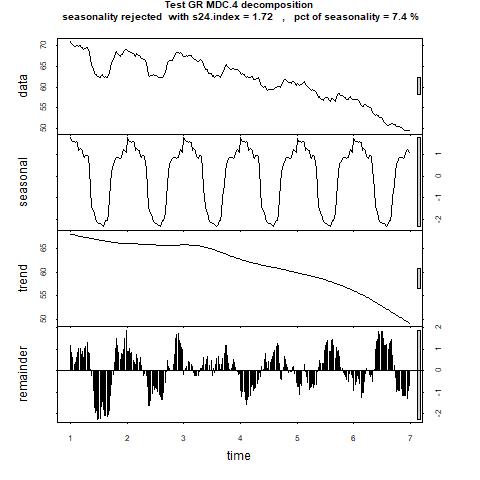

Supplement: Supplementary file 2 — Additional file 2: STL seasonal-trend decomposition for the time series of root electrical capacitance (CR), dissipation factor (DR) and electrical conductance (GR). [file 13007_2023_1133_MOESM2_ESM.zip › GR_MDC4.jpg]

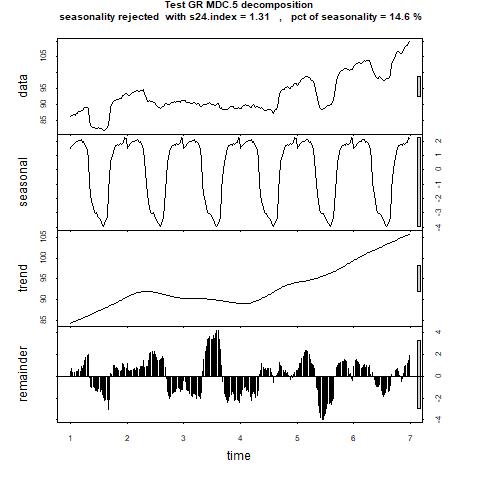

Supplement: Supplementary file 2 — Additional file 2: STL seasonal-trend decomposition for the time series of root electrical capacitance (CR), dissipation factor (DR) and electrical conductance (GR). [file 13007_2023_1133_MOESM2_ESM.zip › GR_MDC5.jpg]

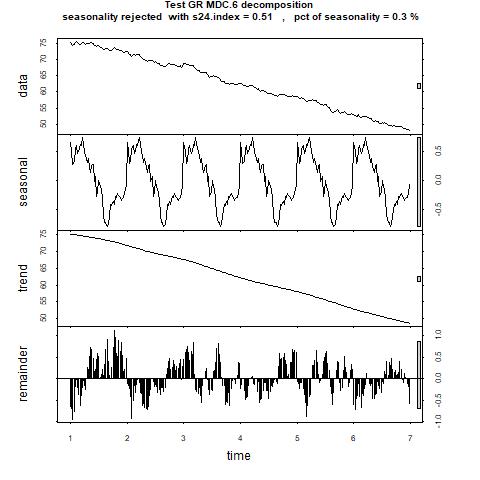

Supplement: Supplementary file 2 — Additional file 2: STL seasonal-trend decomposition for the time series of root electrical capacitance (CR), dissipation factor (DR) and electrical conductance (GR). [file 13007_2023_1133_MOESM2_ESM.zip › GR_MDC6.jpg]

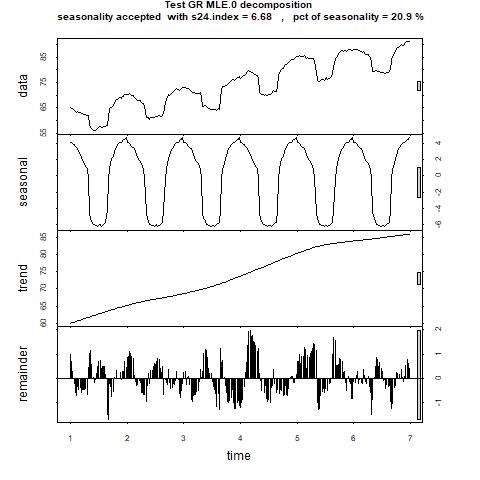

Supplement: Supplementary file 2 — Additional file 2: STL seasonal-trend decomposition for the time series of root electrical capacitance (CR), dissipation factor (DR) and electrical conductance (GR). [file 13007_2023_1133_MOESM2_ESM.zip › GR_MLE0.jpg]

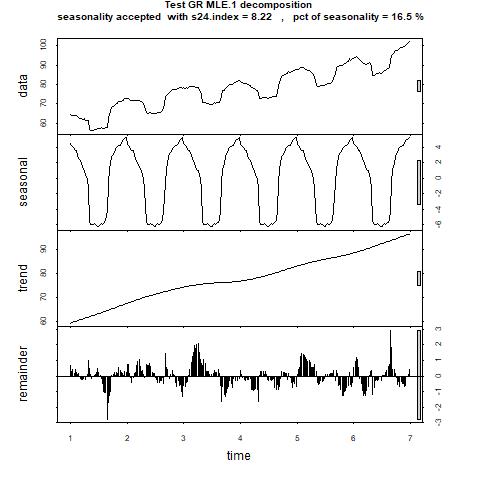

Supplement: Supplementary file 2 — Additional file 2: STL seasonal-trend decomposition for the time series of root electrical capacitance (CR), dissipation factor (DR) and electrical conductance (GR). [file 13007_2023_1133_MOESM2_ESM.zip › GR_MLE1.jpg]

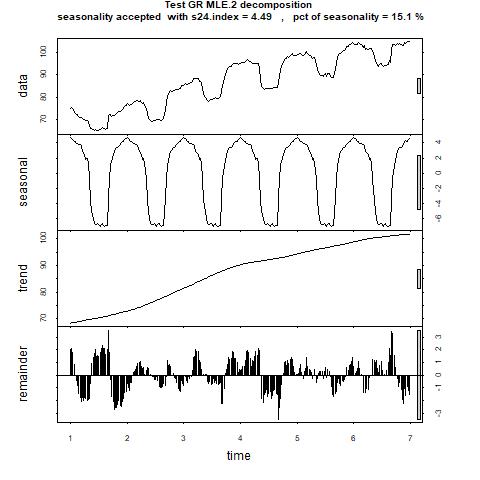

Supplement: Supplementary file 2 — Additional file 2: STL seasonal-trend decomposition for the time series of root electrical capacitance (CR), dissipation factor (DR) and electrical conductance (GR). [file 13007_2023_1133_MOESM2_ESM.zip › GR_MLE2.jpg]

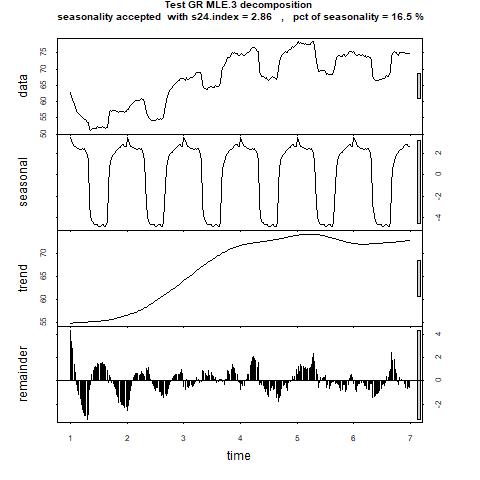

Supplement: Supplementary file 2 — Additional file 2: STL seasonal-trend decomposition for the time series of root electrical capacitance (CR), dissipation factor (DR) and electrical conductance (GR). [file 13007_2023_1133_MOESM2_ESM.zip › GR_MLE3.jpg]

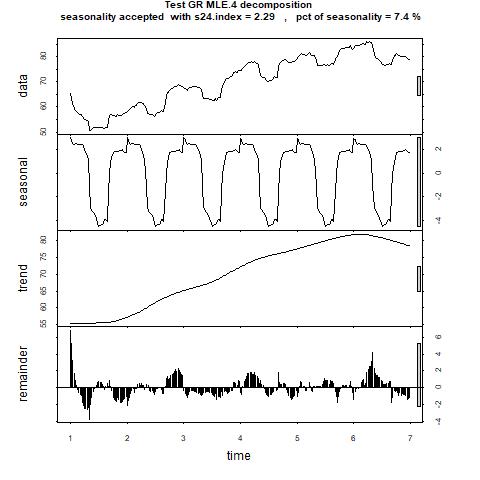

Supplement: Supplementary file 2 — Additional file 2: STL seasonal-trend decomposition for the time series of root electrical capacitance (CR), dissipation factor (DR) and electrical conductance (GR). [file 13007_2023_1133_MOESM2_ESM.zip › GR_MLE4.jpg]

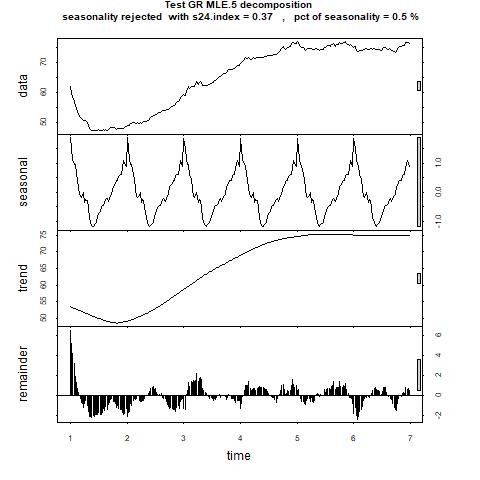

Supplement: Supplementary file 2 — Additional file 2: STL seasonal-trend decomposition for the time series of root electrical capacitance (CR), dissipation factor (DR) and electrical conductance (GR). [file 13007_2023_1133_MOESM2_ESM.zip › GR_MLE5.jpg]

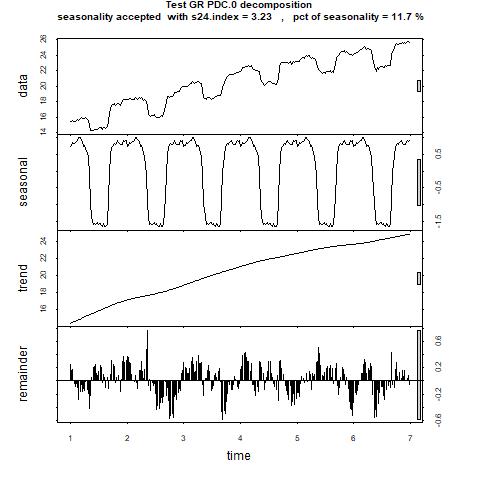

Supplement: Supplementary file 2 — Additional file 2: STL seasonal-trend decomposition for the time series of root electrical capacitance (CR), dissipation factor (DR) and electrical conductance (GR). [file 13007_2023_1133_MOESM2_ESM.zip › GR_PDC0.jpg]

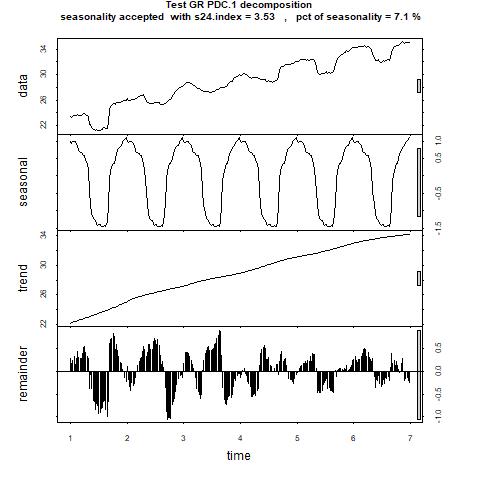

Supplement: Supplementary file 2 — Additional file 2: STL seasonal-trend decomposition for the time series of root electrical capacitance (CR), dissipation factor (DR) and electrical conductance (GR). [file 13007_2023_1133_MOESM2_ESM.zip › GR_PDC1.jpg]

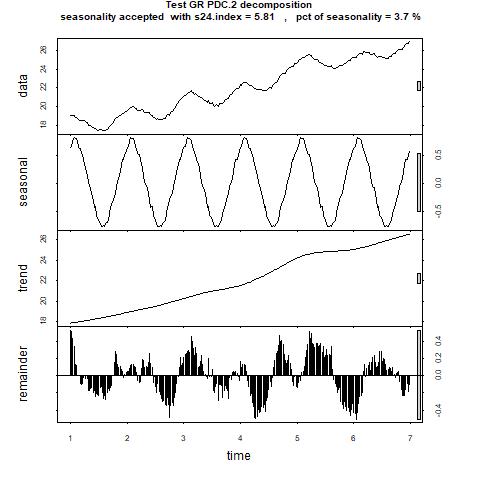

Supplement: Supplementary file 2 — Additional file 2: STL seasonal-trend decomposition for the time series of root electrical capacitance (CR), dissipation factor (DR) and electrical conductance (GR). [file 13007_2023_1133_MOESM2_ESM.zip › GR_PDC2.jpg]

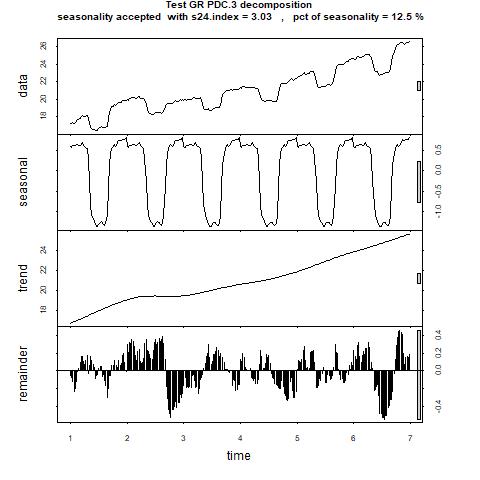

Supplement: Supplementary file 2 — Additional file 2: STL seasonal-trend decomposition for the time series of root electrical capacitance (CR), dissipation factor (DR) and electrical conductance (GR). [file 13007_2023_1133_MOESM2_ESM.zip › GR_PDC3.jpg]

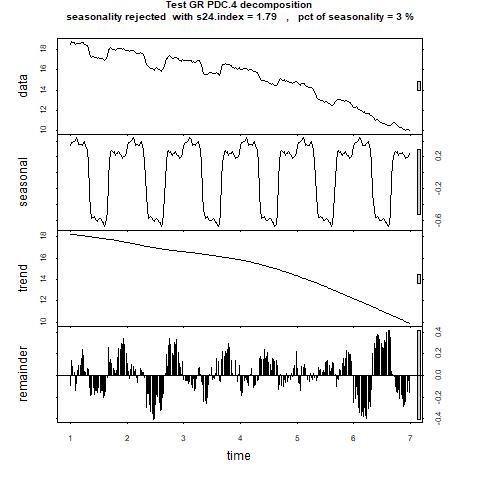

Supplement: Supplementary file 2 — Additional file 2: STL seasonal-trend decomposition for the time series of root electrical capacitance (CR), dissipation factor (DR) and electrical conductance (GR). [file 13007_2023_1133_MOESM2_ESM.zip › GR_PDC4.jpg]

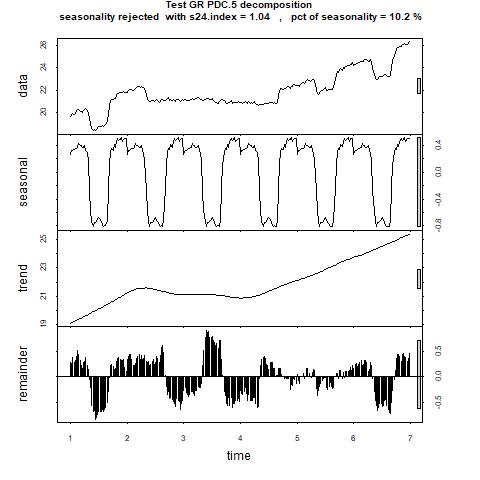

Supplement: Supplementary file 2 — Additional file 2: STL seasonal-trend decomposition for the time series of root electrical capacitance (CR), dissipation factor (DR) and electrical conductance (GR). [file 13007_2023_1133_MOESM2_ESM.zip › GR_PDC5.jpg]

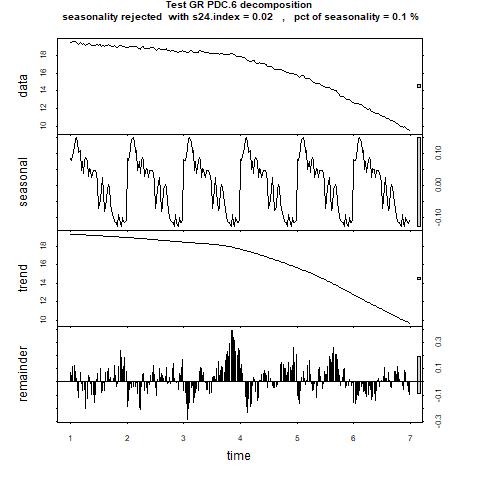

Supplement: Supplementary file 2 — Additional file 2: STL seasonal-trend decomposition for the time series of root electrical capacitance (CR), dissipation factor (DR) and electrical conductance (GR). [file 13007_2023_1133_MOESM2_ESM.zip › GR_PDC6.jpg]

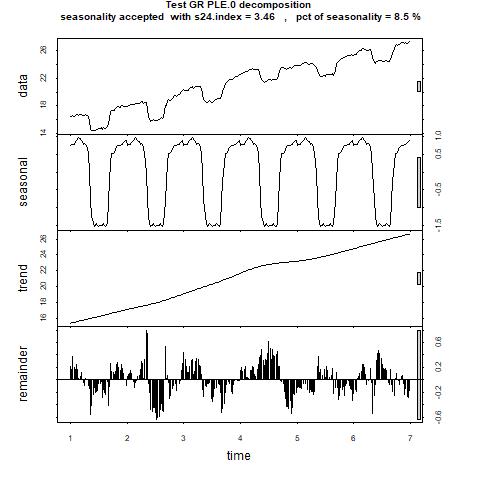

Supplement: Supplementary file 2 — Additional file 2: STL seasonal-trend decomposition for the time series of root electrical capacitance (CR), dissipation factor (DR) and electrical conductance (GR). [file 13007_2023_1133_MOESM2_ESM.zip › GR_PLE0.jpg]

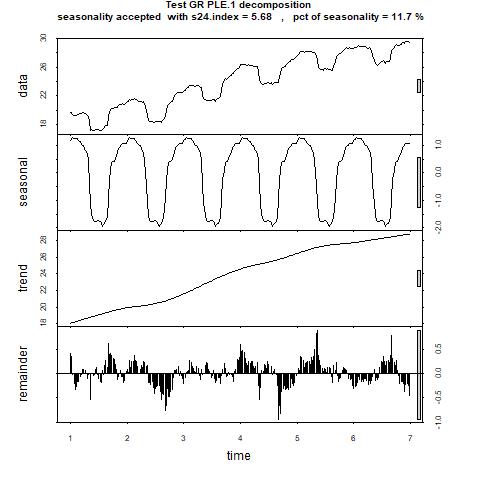

Supplement: Supplementary file 2 — Additional file 2: STL seasonal-trend decomposition for the time series of root electrical capacitance (CR), dissipation factor (DR) and electrical conductance (GR). [file 13007_2023_1133_MOESM2_ESM.zip › GR_PLE1.jpg]

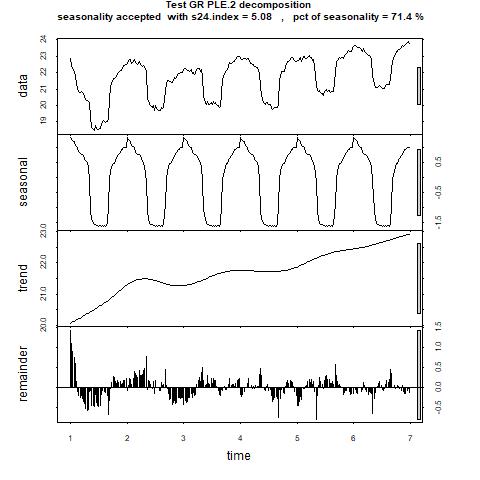

Supplement: Supplementary file 2 — Additional file 2: STL seasonal-trend decomposition for the time series of root electrical capacitance (CR), dissipation factor (DR) and electrical conductance (GR). [file 13007_2023_1133_MOESM2_ESM.zip › GR_PLE2.jpg]

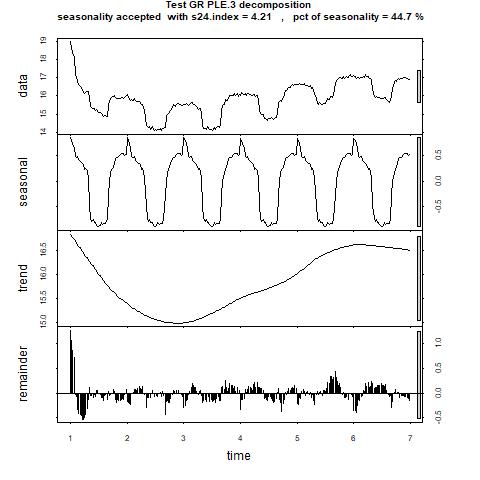

Supplement: Supplementary file 2 — Additional file 2: STL seasonal-trend decomposition for the time series of root electrical capacitance (CR), dissipation factor (DR) and electrical conductance (GR). [file 13007_2023_1133_MOESM2_ESM.zip › GR_PLE3.jpg]

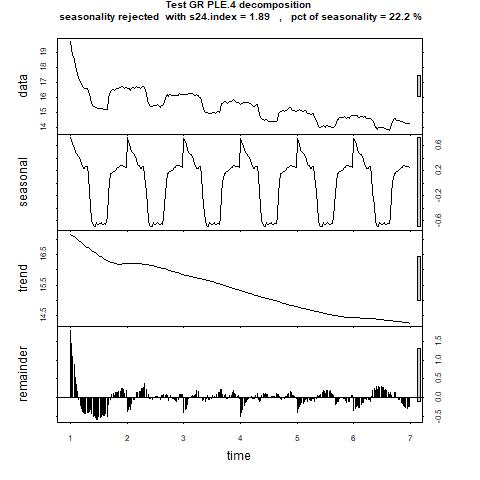

Supplement: Supplementary file 2 — Additional file 2: STL seasonal-trend decomposition for the time series of root electrical capacitance (CR), dissipation factor (DR) and electrical conductance (GR). [file 13007_2023_1133_MOESM2_ESM.zip › GR_PLE4.jpg]

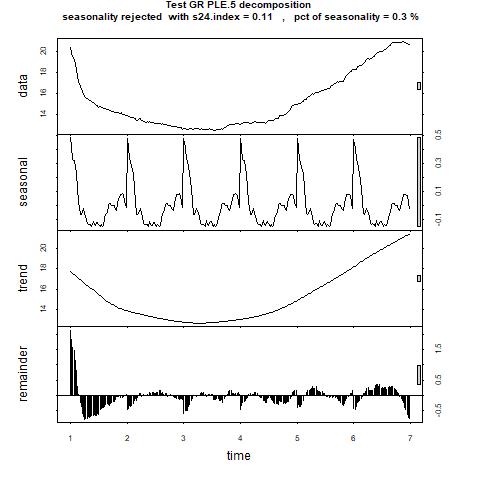

Supplement: Supplementary file 2 — Additional file 2: STL seasonal-trend decomposition for the time series of root electrical capacitance (CR), dissipation factor (DR) and electrical conductance (GR). [file 13007_2023_1133_MOESM2_ESM.zip › GR_PLE5.jpg]

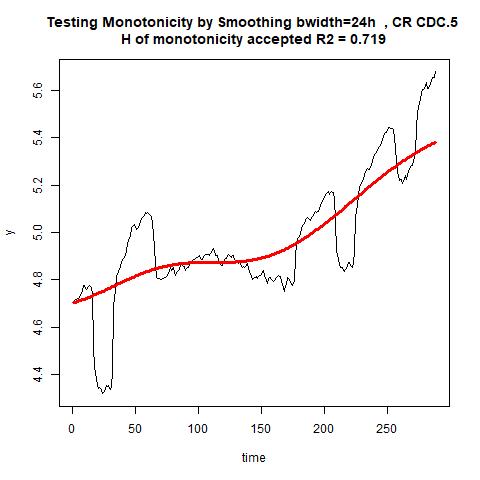

Supplement: Supplementary file 3 — Additional file 3: Monotonicity tests by nonlinear kernel smoothing with up to 24-h bandwidth for the time series of root electrical capacitance (CR), dissipation factor (DR) and electrical conductance (GR). [file 13007_2023_1133_MOESM3_ESM.zip › CR_CDC5.jpg]

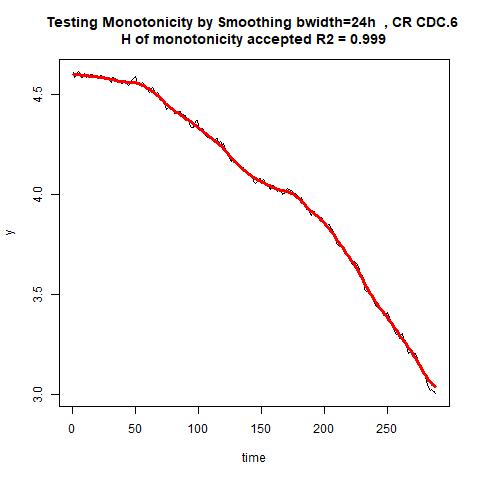

Supplement: Supplementary file 3 — Additional file 3: Monotonicity tests by nonlinear kernel smoothing with up to 24-h bandwidth for the time series of root electrical capacitance (CR), dissipation factor (DR) and electrical conductance (GR). [file 13007_2023_1133_MOESM3_ESM.zip › CR_CDC6.jpg]

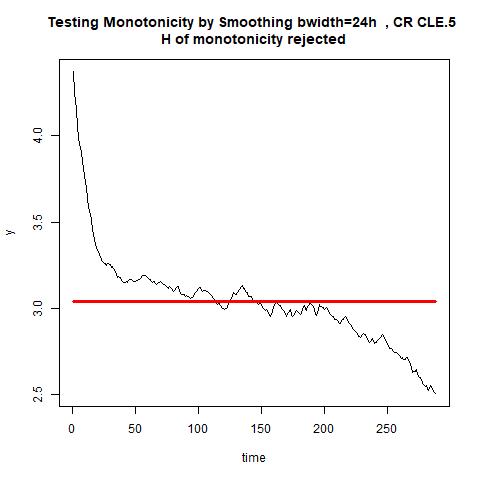

Supplement: Supplementary file 3 — Additional file 3: Monotonicity tests by nonlinear kernel smoothing with up to 24-h bandwidth for the time series of root electrical capacitance (CR), dissipation factor (DR) and electrical conductance (GR). [file 13007_2023_1133_MOESM3_ESM.zip › CR_CLE5.jpg]

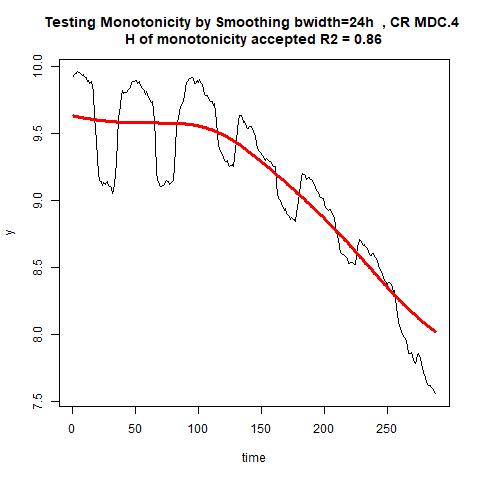

Supplement: Supplementary file 3 — Additional file 3: Monotonicity tests by nonlinear kernel smoothing with up to 24-h bandwidth for the time series of root electrical capacitance (CR), dissipation factor (DR) and electrical conductance (GR). [file 13007_2023_1133_MOESM3_ESM.zip › CR_MDC4.jpg]

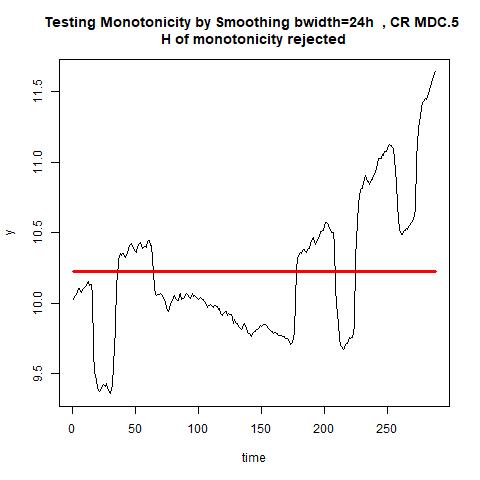

Supplement: Supplementary file 3 — Additional file 3: Monotonicity tests by nonlinear kernel smoothing with up to 24-h bandwidth for the time series of root electrical capacitance (CR), dissipation factor (DR) and electrical conductance (GR). [file 13007_2023_1133_MOESM3_ESM.zip › CR_MDC5.jpg]

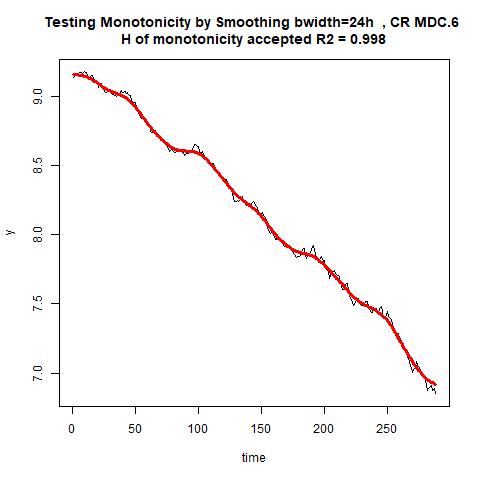

Supplement: Supplementary file 3 — Additional file 3: Monotonicity tests by nonlinear kernel smoothing with up to 24-h bandwidth for the time series of root electrical capacitance (CR), dissipation factor (DR) and electrical conductance (GR). [file 13007_2023_1133_MOESM3_ESM.zip › CR_MDC6.jpg]

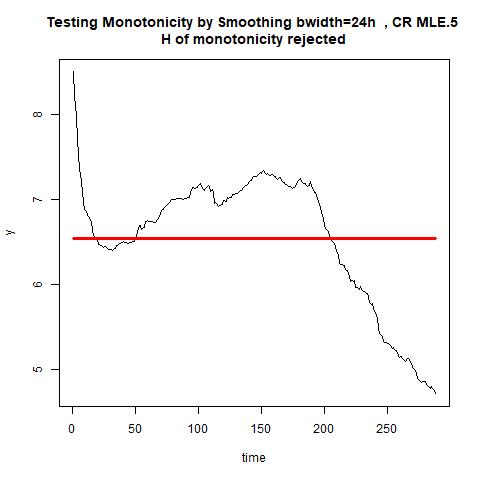

Supplement: Supplementary file 3 — Additional file 3: Monotonicity tests by nonlinear kernel smoothing with up to 24-h bandwidth for the time series of root electrical capacitance (CR), dissipation factor (DR) and electrical conductance (GR). [file 13007_2023_1133_MOESM3_ESM.zip › CR_MLE5.jpg]

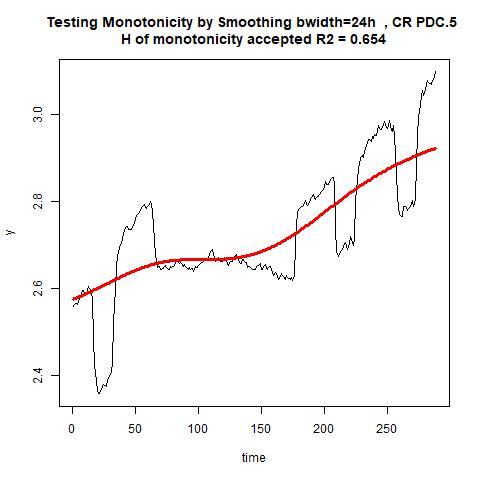

Supplement: Supplementary file 3 — Additional file 3: Monotonicity tests by nonlinear kernel smoothing with up to 24-h bandwidth for the time series of root electrical capacitance (CR), dissipation factor (DR) and electrical conductance (GR). [file 13007_2023_1133_MOESM3_ESM.zip › CR_PDC5.jpg]

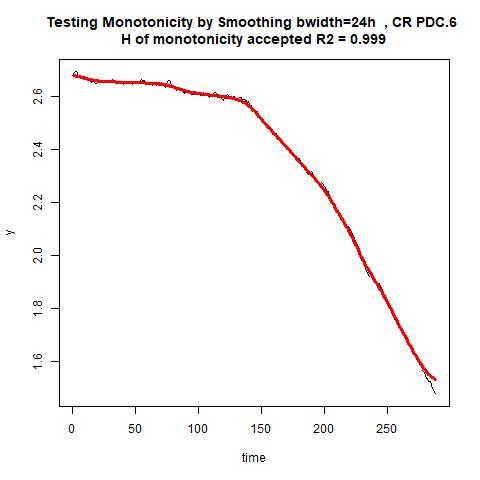

Supplement: Supplementary file 3 — Additional file 3: Monotonicity tests by nonlinear kernel smoothing with up to 24-h bandwidth for the time series of root electrical capacitance (CR), dissipation factor (DR) and electrical conductance (GR). [file 13007_2023_1133_MOESM3_ESM.zip › CR_PDC6.jpg]

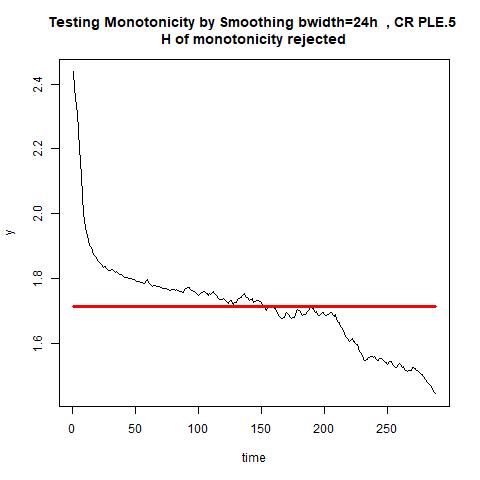

Supplement: Supplementary file 3 — Additional file 3: Monotonicity tests by nonlinear kernel smoothing with up to 24-h bandwidth for the time series of root electrical capacitance (CR), dissipation factor (DR) and electrical conductance (GR). [file 13007_2023_1133_MOESM3_ESM.zip › CR_PLE5.jpg]

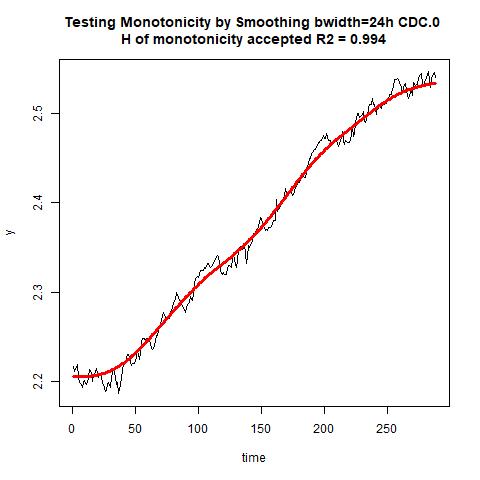

Supplement: Supplementary file 3 — Additional file 3: Monotonicity tests by nonlinear kernel smoothing with up to 24-h bandwidth for the time series of root electrical capacitance (CR), dissipation factor (DR) and electrical conductance (GR). [file 13007_2023_1133_MOESM3_ESM.zip › DR_CDC0.jpg]

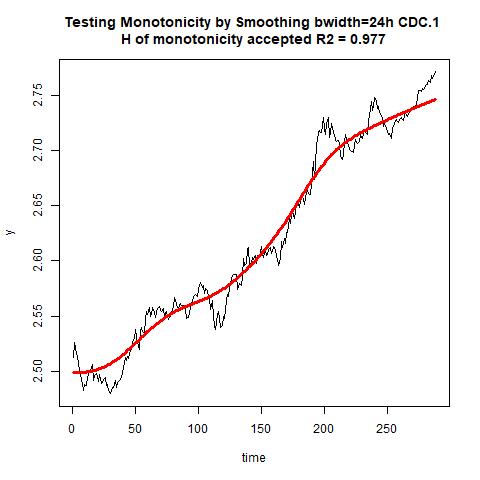

Supplement: Supplementary file 3 — Additional file 3: Monotonicity tests by nonlinear kernel smoothing with up to 24-h bandwidth for the time series of root electrical capacitance (CR), dissipation factor (DR) and electrical conductance (GR). [file 13007_2023_1133_MOESM3_ESM.zip › DR_CDC1.jpg]

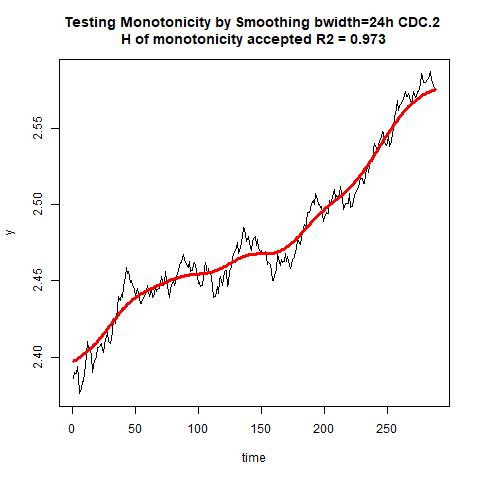

Supplement: Supplementary file 3 — Additional file 3: Monotonicity tests by nonlinear kernel smoothing with up to 24-h bandwidth for the time series of root electrical capacitance (CR), dissipation factor (DR) and electrical conductance (GR). [file 13007_2023_1133_MOESM3_ESM.zip › DR_CDC2.jpg]

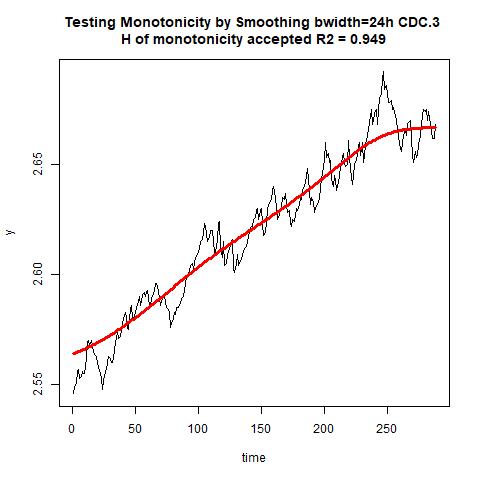

Supplement: Supplementary file 3 — Additional file 3: Monotonicity tests by nonlinear kernel smoothing with up to 24-h bandwidth for the time series of root electrical capacitance (CR), dissipation factor (DR) and electrical conductance (GR). [file 13007_2023_1133_MOESM3_ESM.zip › DR_CDC3.jpg]

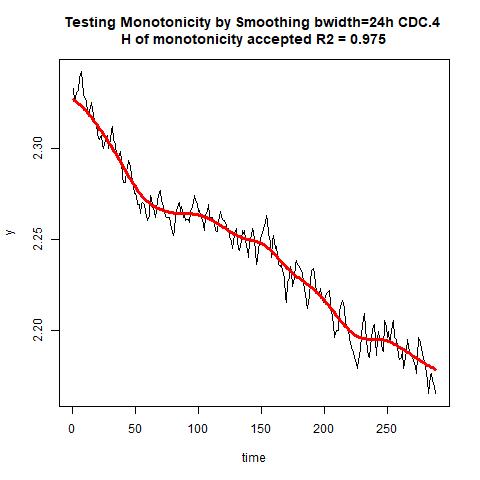

Supplement: Supplementary file 3 — Additional file 3: Monotonicity tests by nonlinear kernel smoothing with up to 24-h bandwidth for the time series of root electrical capacitance (CR), dissipation factor (DR) and electrical conductance (GR). [file 13007_2023_1133_MOESM3_ESM.zip › DR_CDC4.jpg]

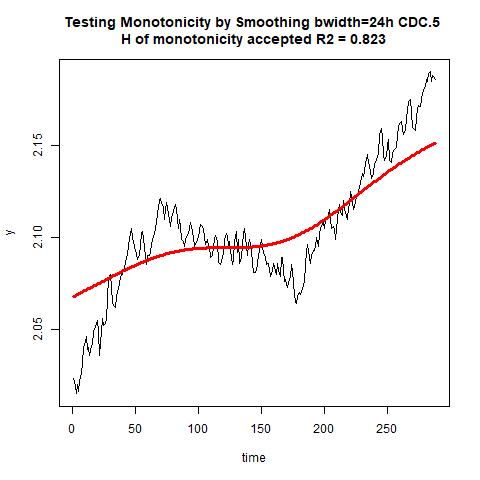

Supplement: Supplementary file 3 — Additional file 3: Monotonicity tests by nonlinear kernel smoothing with up to 24-h bandwidth for the time series of root electrical capacitance (CR), dissipation factor (DR) and electrical conductance (GR). [file 13007_2023_1133_MOESM3_ESM.zip › DR_CDC5.jpg]

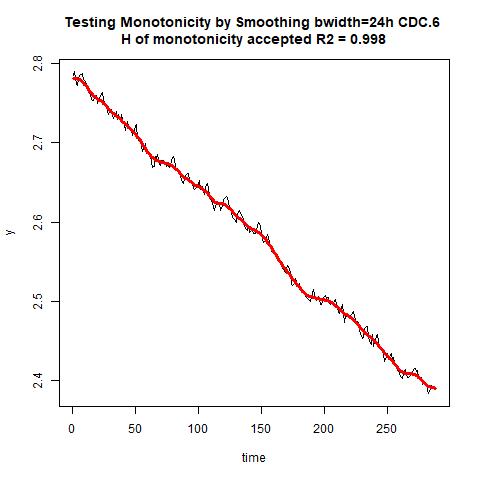

Supplement: Supplementary file 3 — Additional file 3: Monotonicity tests by nonlinear kernel smoothing with up to 24-h bandwidth for the time series of root electrical capacitance (CR), dissipation factor (DR) and electrical conductance (GR). [file 13007_2023_1133_MOESM3_ESM.zip › DR_CDC6.jpg]

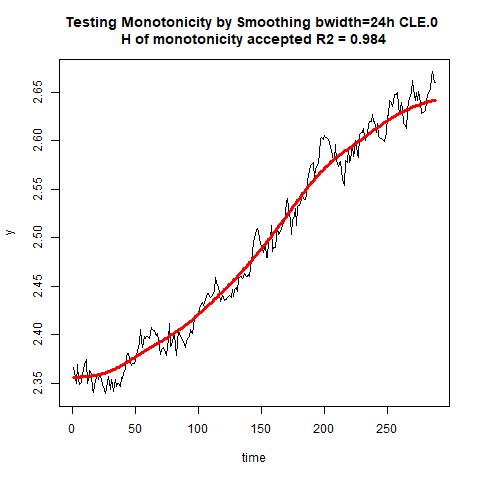

Supplement: Supplementary file 3 — Additional file 3: Monotonicity tests by nonlinear kernel smoothing with up to 24-h bandwidth for the time series of root electrical capacitance (CR), dissipation factor (DR) and electrical conductance (GR). [file 13007_2023_1133_MOESM3_ESM.zip › DR_CLE0.jpg]

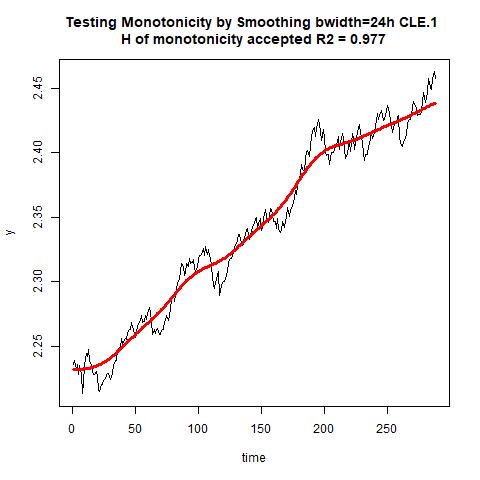

Supplement: Supplementary file 3 — Additional file 3: Monotonicity tests by nonlinear kernel smoothing with up to 24-h bandwidth for the time series of root electrical capacitance (CR), dissipation factor (DR) and electrical conductance (GR). [file 13007_2023_1133_MOESM3_ESM.zip › DR_CLE1.jpg]

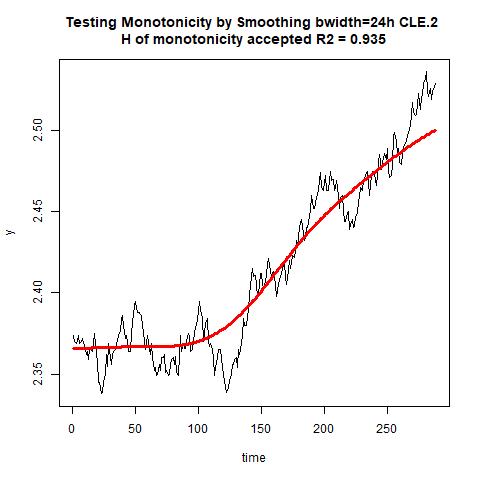

Supplement: Supplementary file 3 — Additional file 3: Monotonicity tests by nonlinear kernel smoothing with up to 24-h bandwidth for the time series of root electrical capacitance (CR), dissipation factor (DR) and electrical conductance (GR). [file 13007_2023_1133_MOESM3_ESM.zip › DR_CLE2.jpg]

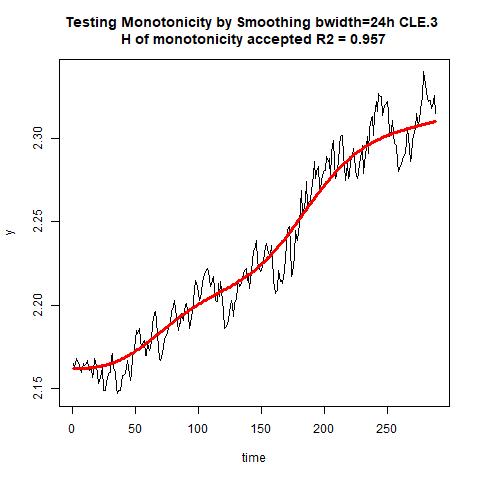

Supplement: Supplementary file 3 — Additional file 3: Monotonicity tests by nonlinear kernel smoothing with up to 24-h bandwidth for the time series of root electrical capacitance (CR), dissipation factor (DR) and electrical conductance (GR). [file 13007_2023_1133_MOESM3_ESM.zip › DR_CLE3.jpg]

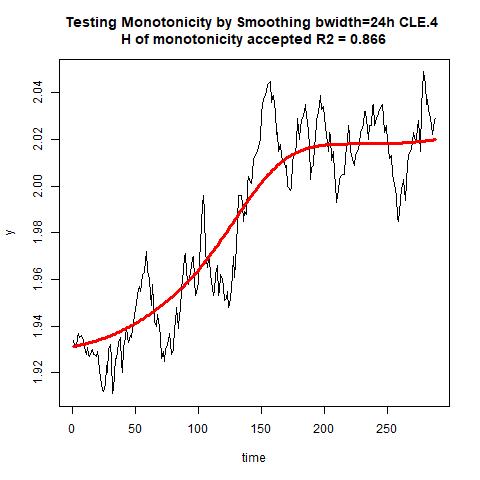

Supplement: Supplementary file 3 — Additional file 3: Monotonicity tests by nonlinear kernel smoothing with up to 24-h bandwidth for the time series of root electrical capacitance (CR), dissipation factor (DR) and electrical conductance (GR). [file 13007_2023_1133_MOESM3_ESM.zip › DR_CLE4.jpg]

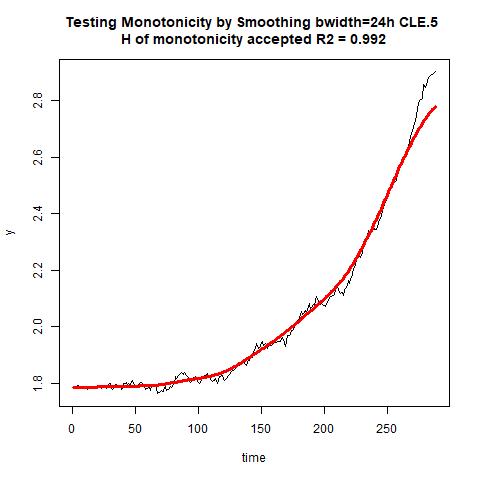

Supplement: Supplementary file 3 — Additional file 3: Monotonicity tests by nonlinear kernel smoothing with up to 24-h bandwidth for the time series of root electrical capacitance (CR), dissipation factor (DR) and electrical conductance (GR). [file 13007_2023_1133_MOESM3_ESM.zip › DR_CLE5.jpg]

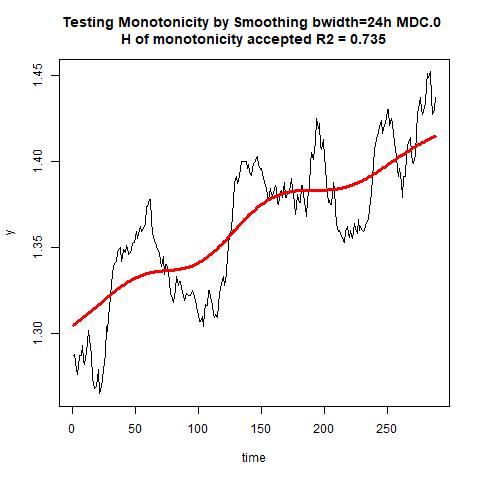

Supplement: Supplementary file 3 — Additional file 3: Monotonicity tests by nonlinear kernel smoothing with up to 24-h bandwidth for the time series of root electrical capacitance (CR), dissipation factor (DR) and electrical conductance (GR). [file 13007_2023_1133_MOESM3_ESM.zip › DR_MDC0.jpg]

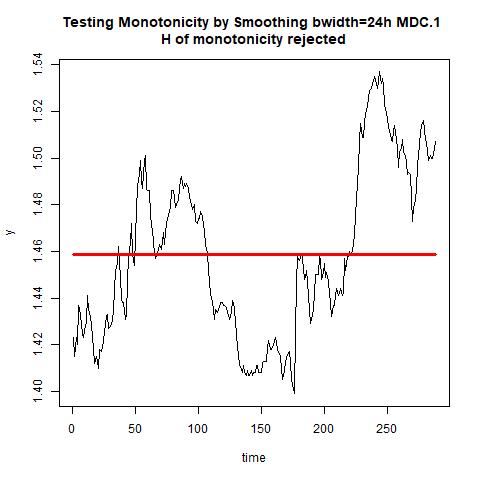

Supplement: Supplementary file 3 — Additional file 3: Monotonicity tests by nonlinear kernel smoothing with up to 24-h bandwidth for the time series of root electrical capacitance (CR), dissipation factor (DR) and electrical conductance (GR). [file 13007_2023_1133_MOESM3_ESM.zip › DR_MDC1.jpg]

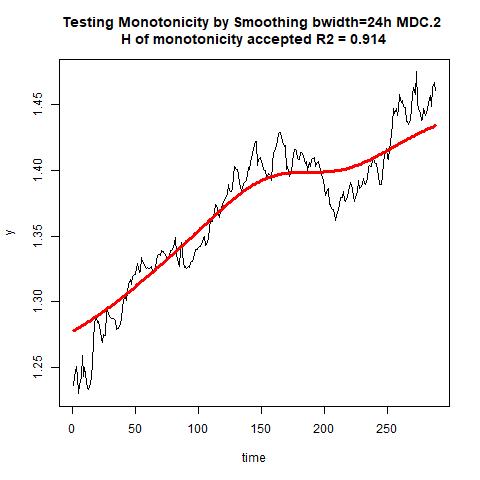

Supplement: Supplementary file 3 — Additional file 3: Monotonicity tests by nonlinear kernel smoothing with up to 24-h bandwidth for the time series of root electrical capacitance (CR), dissipation factor (DR) and electrical conductance (GR). [file 13007_2023_1133_MOESM3_ESM.zip › DR_MDC2.jpg]

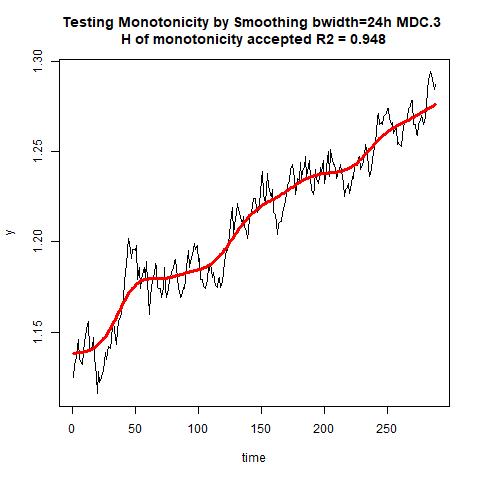

Supplement: Supplementary file 3 — Additional file 3: Monotonicity tests by nonlinear kernel smoothing with up to 24-h bandwidth for the time series of root electrical capacitance (CR), dissipation factor (DR) and electrical conductance (GR). [file 13007_2023_1133_MOESM3_ESM.zip › DR_MDC3.jpg]

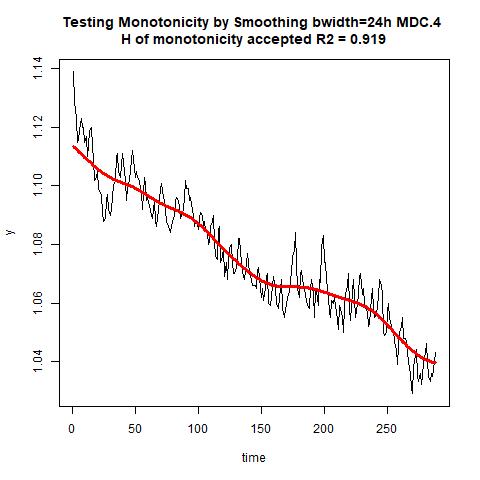

Supplement: Supplementary file 3 — Additional file 3: Monotonicity tests by nonlinear kernel smoothing with up to 24-h bandwidth for the time series of root electrical capacitance (CR), dissipation factor (DR) and electrical conductance (GR). [file 13007_2023_1133_MOESM3_ESM.zip › DR_MDC4.jpg]

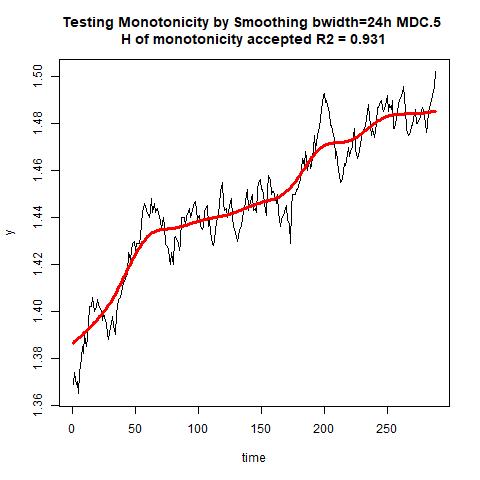

Supplement: Supplementary file 3 — Additional file 3: Monotonicity tests by nonlinear kernel smoothing with up to 24-h bandwidth for the time series of root electrical capacitance (CR), dissipation factor (DR) and electrical conductance (GR). [file 13007_2023_1133_MOESM3_ESM.zip › DR_MDC5.jpg]

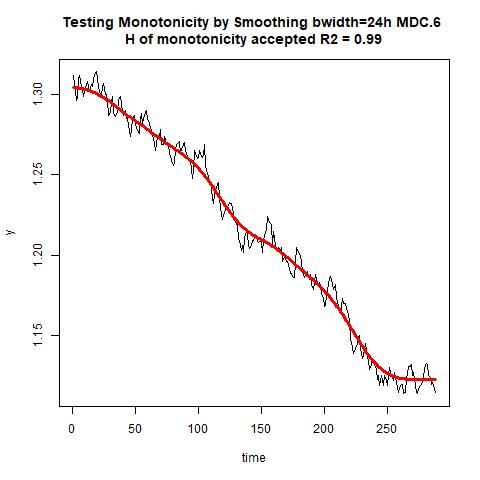

Supplement: Supplementary file 3 — Additional file 3: Monotonicity tests by nonlinear kernel smoothing with up to 24-h bandwidth for the time series of root electrical capacitance (CR), dissipation factor (DR) and electrical conductance (GR). [file 13007_2023_1133_MOESM3_ESM.zip › DR_MDC6.jpg]

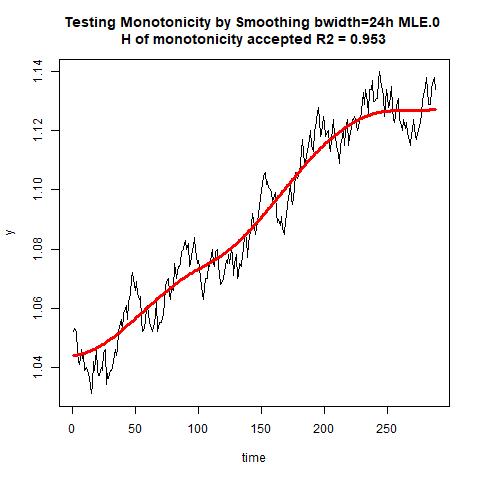

Supplement: Supplementary file 3 — Additional file 3: Monotonicity tests by nonlinear kernel smoothing with up to 24-h bandwidth for the time series of root electrical capacitance (CR), dissipation factor (DR) and electrical conductance (GR). [file 13007_2023_1133_MOESM3_ESM.zip › DR_MLE0.jpg]

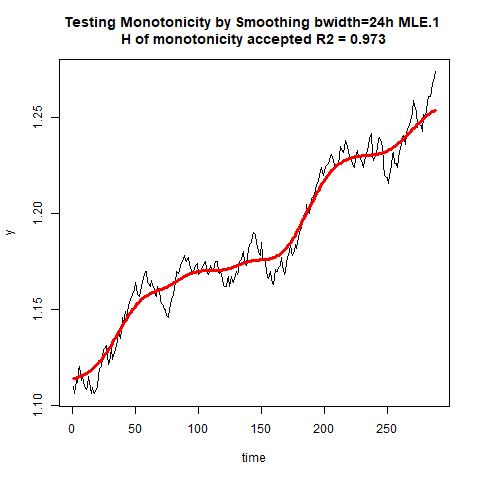

Supplement: Supplementary file 3 — Additional file 3: Monotonicity tests by nonlinear kernel smoothing with up to 24-h bandwidth for the time series of root electrical capacitance (CR), dissipation factor (DR) and electrical conductance (GR). [file 13007_2023_1133_MOESM3_ESM.zip › DR_MLE1.jpg]

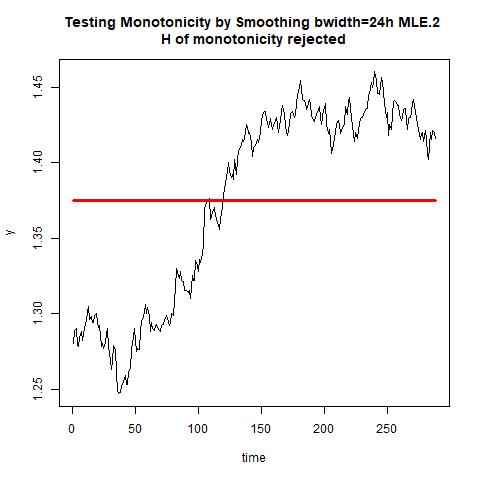

Supplement: Supplementary file 3 — Additional file 3: Monotonicity tests by nonlinear kernel smoothing with up to 24-h bandwidth for the time series of root electrical capacitance (CR), dissipation factor (DR) and electrical conductance (GR). [file 13007_2023_1133_MOESM3_ESM.zip › DR_MLE2.jpg]

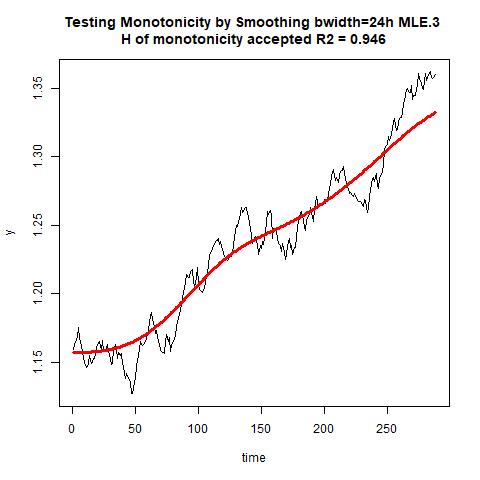

Supplement: Supplementary file 3 — Additional file 3: Monotonicity tests by nonlinear kernel smoothing with up to 24-h bandwidth for the time series of root electrical capacitance (CR), dissipation factor (DR) and electrical conductance (GR). [file 13007_2023_1133_MOESM3_ESM.zip › DR_MLE3.jpg]

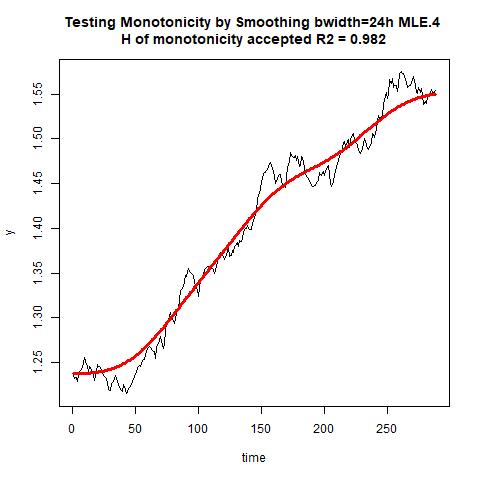

Supplement: Supplementary file 3 — Additional file 3: Monotonicity tests by nonlinear kernel smoothing with up to 24-h bandwidth for the time series of root electrical capacitance (CR), dissipation factor (DR) and electrical conductance (GR). [file 13007_2023_1133_MOESM3_ESM.zip › DR_MLE4.jpg]

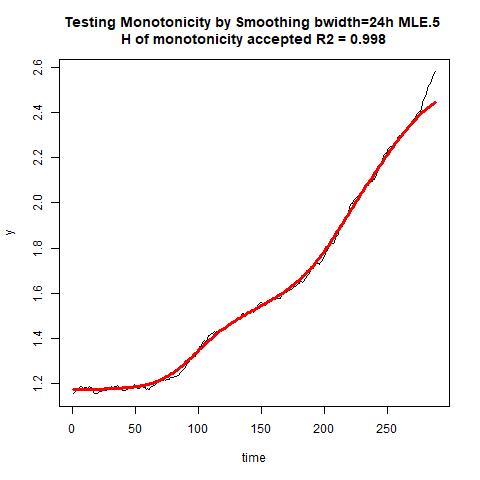

Supplement: Supplementary file 3 — Additional file 3: Monotonicity tests by nonlinear kernel smoothing with up to 24-h bandwidth for the time series of root electrical capacitance (CR), dissipation factor (DR) and electrical conductance (GR). [file 13007_2023_1133_MOESM3_ESM.zip › DR_MLE5.jpg]

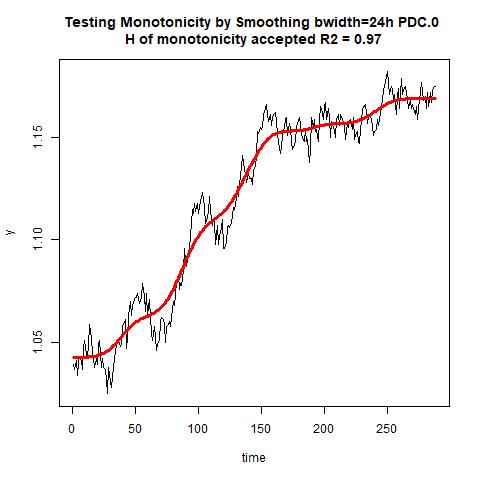

Supplement: Supplementary file 3 — Additional file 3: Monotonicity tests by nonlinear kernel smoothing with up to 24-h bandwidth for the time series of root electrical capacitance (CR), dissipation factor (DR) and electrical conductance (GR). [file 13007_2023_1133_MOESM3_ESM.zip › DR_PDC0.jpg]

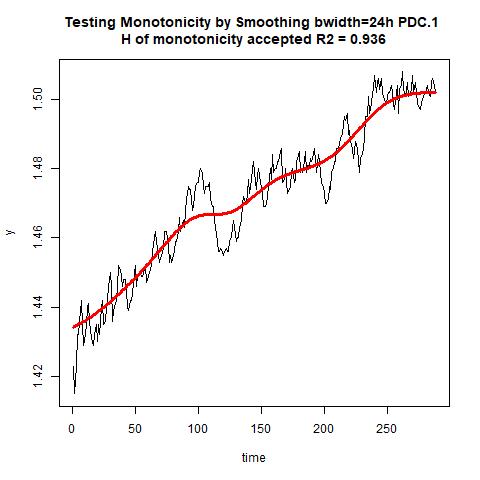

Supplement: Supplementary file 3 — Additional file 3: Monotonicity tests by nonlinear kernel smoothing with up to 24-h bandwidth for the time series of root electrical capacitance (CR), dissipation factor (DR) and electrical conductance (GR). [file 13007_2023_1133_MOESM3_ESM.zip › DR_PDC1.jpg]

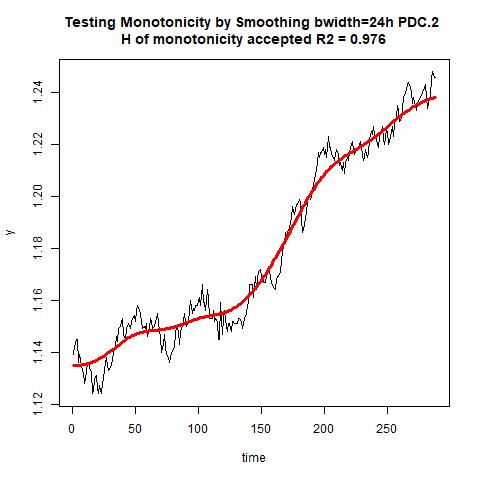

Supplement: Supplementary file 3 — Additional file 3: Monotonicity tests by nonlinear kernel smoothing with up to 24-h bandwidth for the time series of root electrical capacitance (CR), dissipation factor (DR) and electrical conductance (GR). [file 13007_2023_1133_MOESM3_ESM.zip › DR_PDC2.jpg]

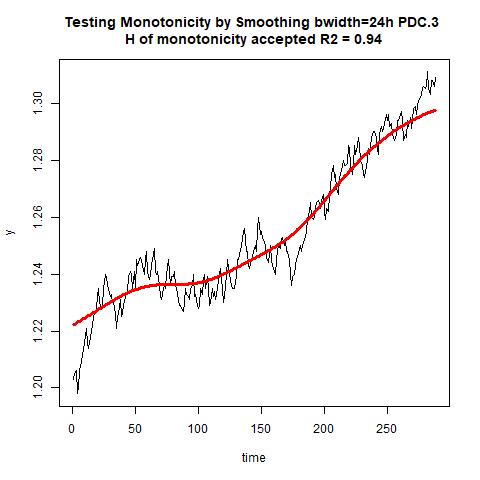

Supplement: Supplementary file 3 — Additional file 3: Monotonicity tests by nonlinear kernel smoothing with up to 24-h bandwidth for the time series of root electrical capacitance (CR), dissipation factor (DR) and electrical conductance (GR). [file 13007_2023_1133_MOESM3_ESM.zip › DR_PDC3.jpg]

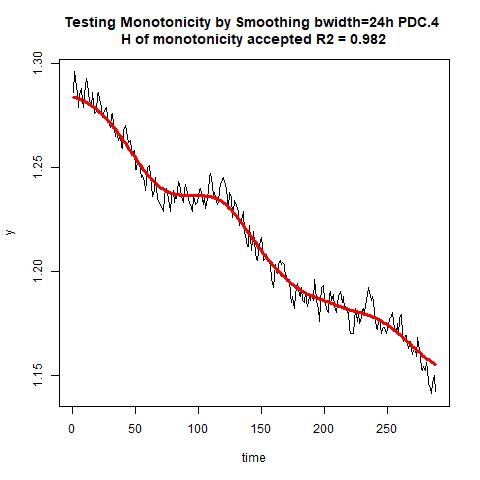

Supplement: Supplementary file 3 — Additional file 3: Monotonicity tests by nonlinear kernel smoothing with up to 24-h bandwidth for the time series of root electrical capacitance (CR), dissipation factor (DR) and electrical conductance (GR). [file 13007_2023_1133_MOESM3_ESM.zip › DR_PDC4.jpg]

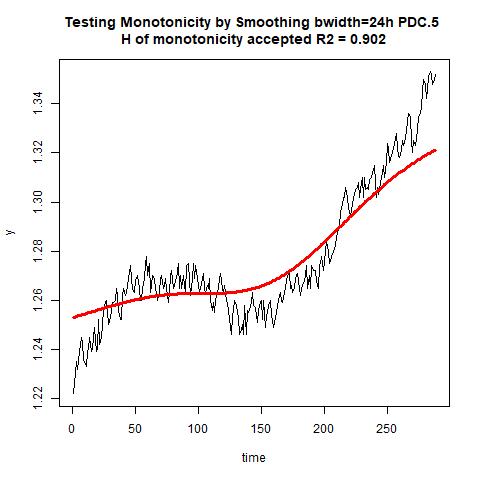

Supplement: Supplementary file 3 — Additional file 3: Monotonicity tests by nonlinear kernel smoothing with up to 24-h bandwidth for the time series of root electrical capacitance (CR), dissipation factor (DR) and electrical conductance (GR). [file 13007_2023_1133_MOESM3_ESM.zip › DR_PDC5.jpg]

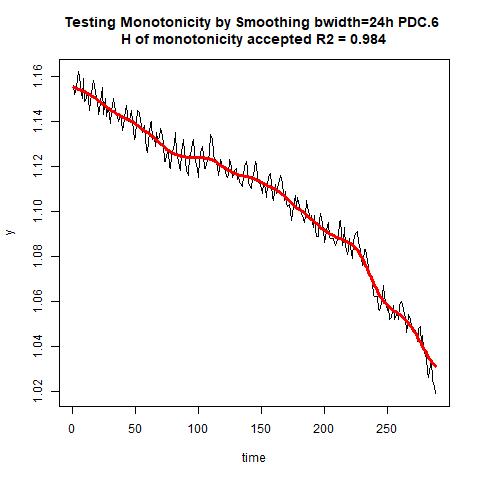

Supplement: Supplementary file 3 — Additional file 3: Monotonicity tests by nonlinear kernel smoothing with up to 24-h bandwidth for the time series of root electrical capacitance (CR), dissipation factor (DR) and electrical conductance (GR). [file 13007_2023_1133_MOESM3_ESM.zip › DR_PDC6.jpg]

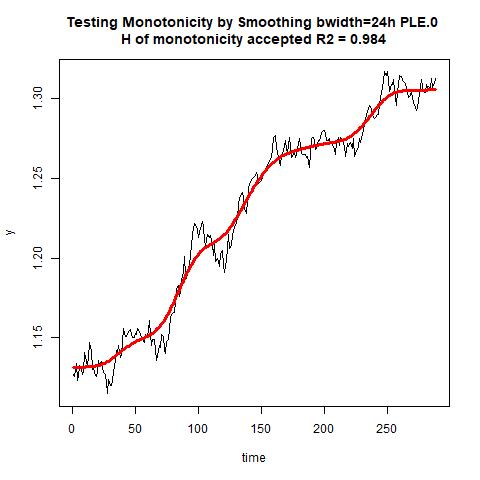

Supplement: Supplementary file 3 — Additional file 3: Monotonicity tests by nonlinear kernel smoothing with up to 24-h bandwidth for the time series of root electrical capacitance (CR), dissipation factor (DR) and electrical conductance (GR). [file 13007_2023_1133_MOESM3_ESM.zip › DR_PLE0.jpg]

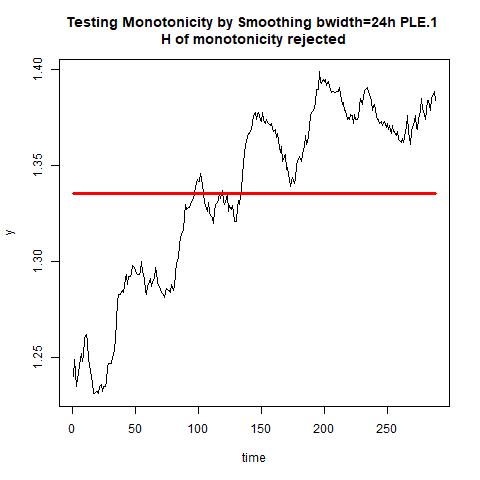

Supplement: Supplementary file 3 — Additional file 3: Monotonicity tests by nonlinear kernel smoothing with up to 24-h bandwidth for the time series of root electrical capacitance (CR), dissipation factor (DR) and electrical conductance (GR). [file 13007_2023_1133_MOESM3_ESM.zip › DR_PLE1.jpg]

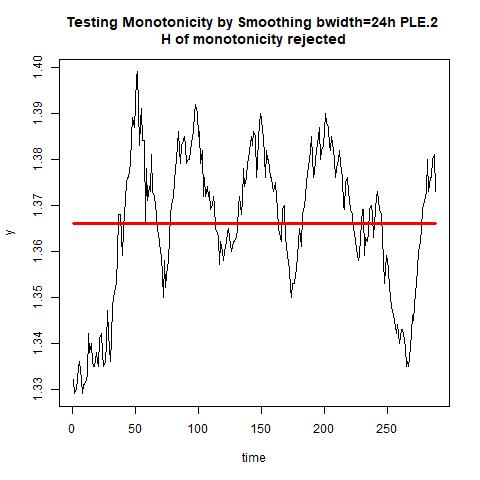

Supplement: Supplementary file 3 — Additional file 3: Monotonicity tests by nonlinear kernel smoothing with up to 24-h bandwidth for the time series of root electrical capacitance (CR), dissipation factor (DR) and electrical conductance (GR). [file 13007_2023_1133_MOESM3_ESM.zip › DR_PLE2.jpg]

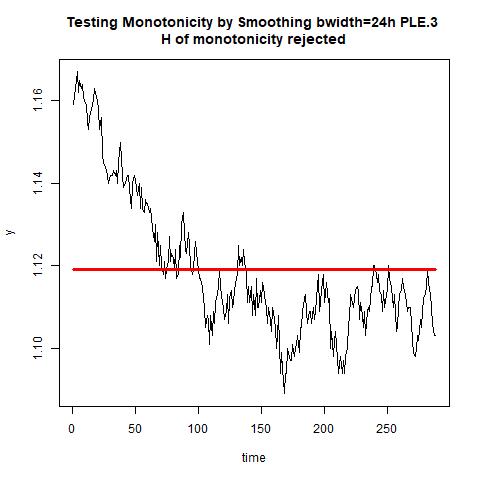

Supplement: Supplementary file 3 — Additional file 3: Monotonicity tests by nonlinear kernel smoothing with up to 24-h bandwidth for the time series of root electrical capacitance (CR), dissipation factor (DR) and electrical conductance (GR). [file 13007_2023_1133_MOESM3_ESM.zip › DR_PLE3.jpg]

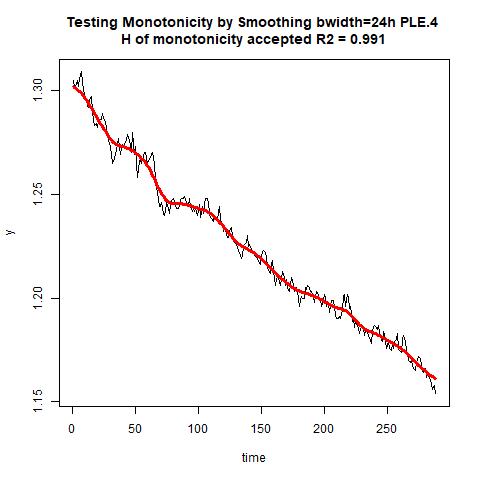

Supplement: Supplementary file 3 — Additional file 3: Monotonicity tests by nonlinear kernel smoothing with up to 24-h bandwidth for the time series of root electrical capacitance (CR), dissipation factor (DR) and electrical conductance (GR). [file 13007_2023_1133_MOESM3_ESM.zip › DR_PLE4.jpg]

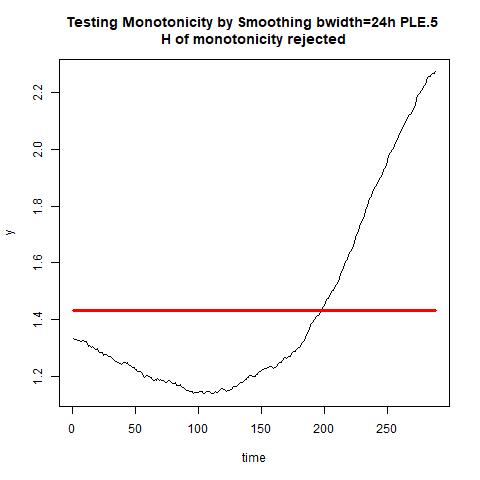

Supplement: Supplementary file 3 — Additional file 3: Monotonicity tests by nonlinear kernel smoothing with up to 24-h bandwidth for the time series of root electrical capacitance (CR), dissipation factor (DR) and electrical conductance (GR). [file 13007_2023_1133_MOESM3_ESM.zip › DR_PLE5.jpg]

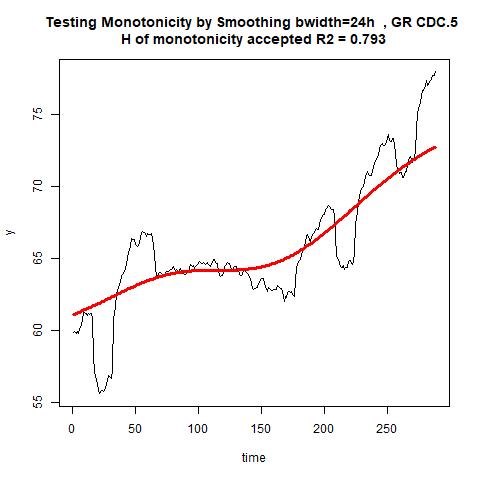

Supplement: Supplementary file 3 — Additional file 3: Monotonicity tests by nonlinear kernel smoothing with up to 24-h bandwidth for the time series of root electrical capacitance (CR), dissipation factor (DR) and electrical conductance (GR). [file 13007_2023_1133_MOESM3_ESM.zip › GR_CDC5.jpg]

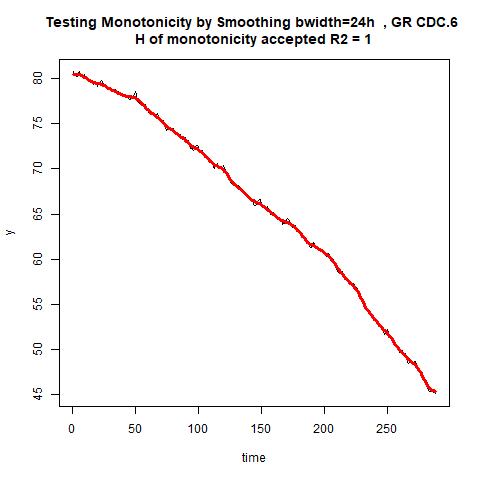

Supplement: Supplementary file 3 — Additional file 3: Monotonicity tests by nonlinear kernel smoothing with up to 24-h bandwidth for the time series of root electrical capacitance (CR), dissipation factor (DR) and electrical conductance (GR). [file 13007_2023_1133_MOESM3_ESM.zip › GR_CDC6.jpg]

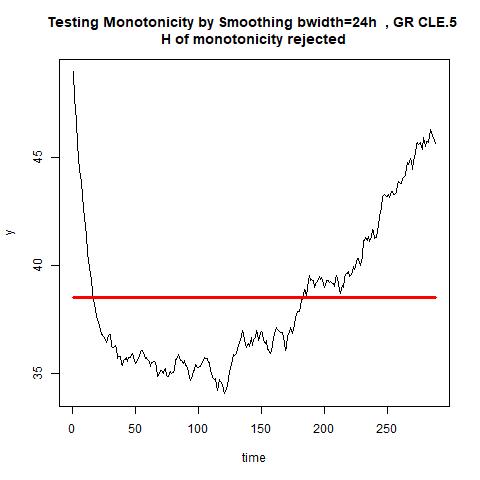

Supplement: Supplementary file 3 — Additional file 3: Monotonicity tests by nonlinear kernel smoothing with up to 24-h bandwidth for the time series of root electrical capacitance (CR), dissipation factor (DR) and electrical conductance (GR). [file 13007_2023_1133_MOESM3_ESM.zip › GR_CLE5.jpg]

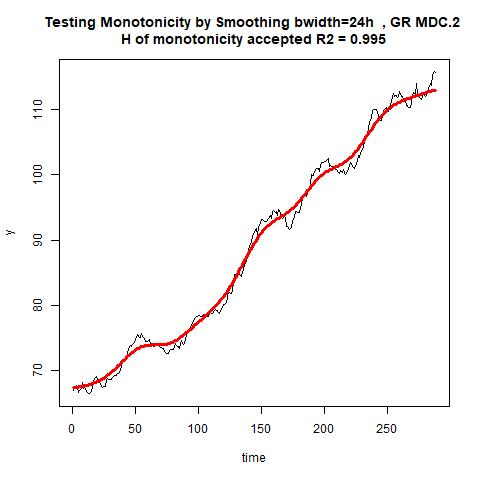

Supplement: Supplementary file 3 — Additional file 3: Monotonicity tests by nonlinear kernel smoothing with up to 24-h bandwidth for the time series of root electrical capacitance (CR), dissipation factor (DR) and electrical conductance (GR). [file 13007_2023_1133_MOESM3_ESM.zip › GR_MDC2.jpg]

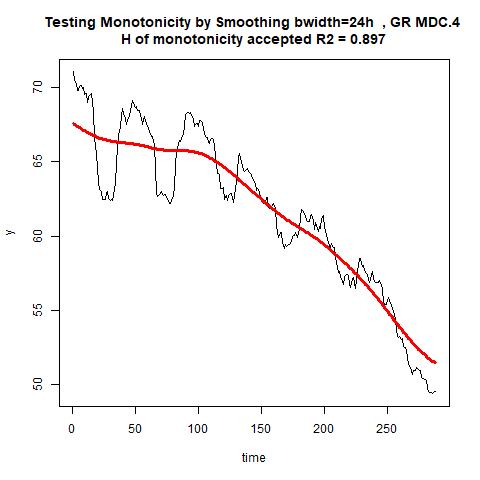

Supplement: Supplementary file 3 — Additional file 3: Monotonicity tests by nonlinear kernel smoothing with up to 24-h bandwidth for the time series of root electrical capacitance (CR), dissipation factor (DR) and electrical conductance (GR). [file 13007_2023_1133_MOESM3_ESM.zip › GR_MDC4.jpg]

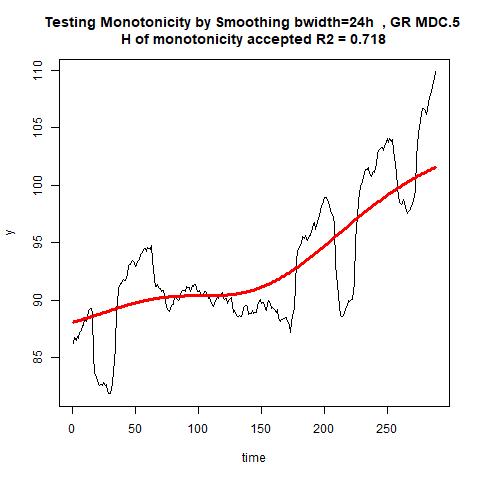

Supplement: Supplementary file 3 — Additional file 3: Monotonicity tests by nonlinear kernel smoothing with up to 24-h bandwidth for the time series of root electrical capacitance (CR), dissipation factor (DR) and electrical conductance (GR). [file 13007_2023_1133_MOESM3_ESM.zip › GR_MDC5.jpg]

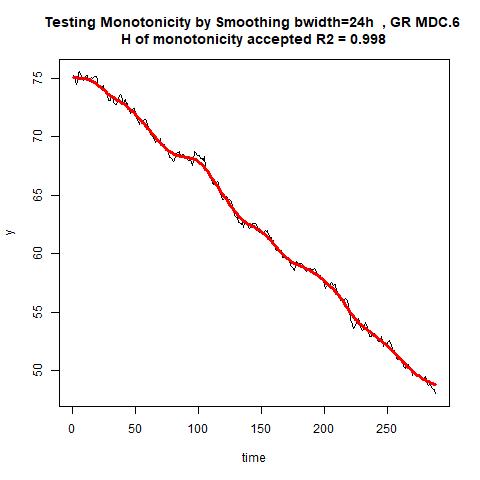

Supplement: Supplementary file 3 — Additional file 3: Monotonicity tests by nonlinear kernel smoothing with up to 24-h bandwidth for the time series of root electrical capacitance (CR), dissipation factor (DR) and electrical conductance (GR). [file 13007_2023_1133_MOESM3_ESM.zip › GR_MDC6.jpg]

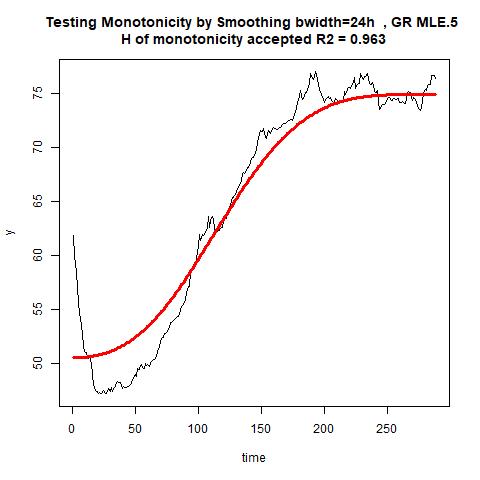

Supplement: Supplementary file 3 — Additional file 3: Monotonicity tests by nonlinear kernel smoothing with up to 24-h bandwidth for the time series of root electrical capacitance (CR), dissipation factor (DR) and electrical conductance (GR). [file 13007_2023_1133_MOESM3_ESM.zip › GR_MLE5.jpg]

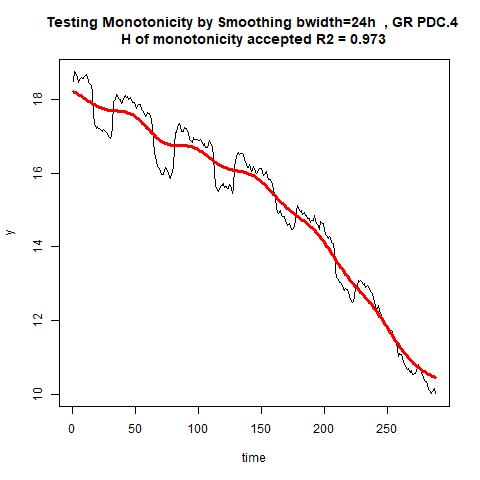

Supplement: Supplementary file 3 — Additional file 3: Monotonicity tests by nonlinear kernel smoothing with up to 24-h bandwidth for the time series of root electrical capacitance (CR), dissipation factor (DR) and electrical conductance (GR). [file 13007_2023_1133_MOESM3_ESM.zip › GR_PDC4.jpg]

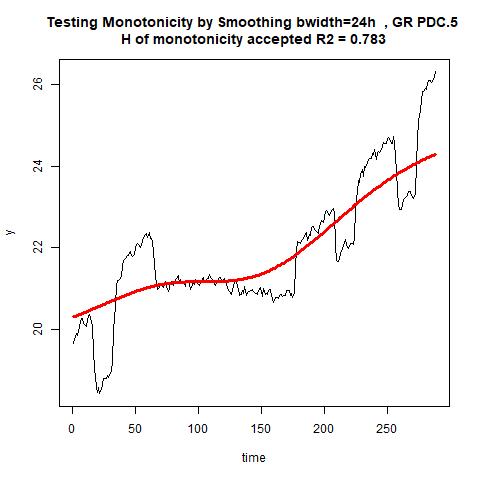

Supplement: Supplementary file 3 — Additional file 3: Monotonicity tests by nonlinear kernel smoothing with up to 24-h bandwidth for the time series of root electrical capacitance (CR), dissipation factor (DR) and electrical conductance (GR). [file 13007_2023_1133_MOESM3_ESM.zip › GR_PDC5.jpg]

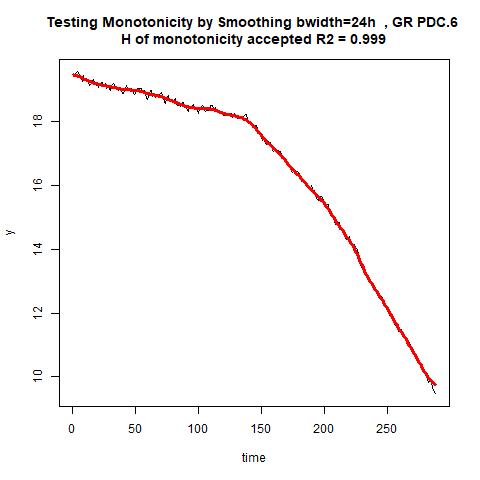

Supplement: Supplementary file 3 — Additional file 3: Monotonicity tests by nonlinear kernel smoothing with up to 24-h bandwidth for the time series of root electrical capacitance (CR), dissipation factor (DR) and electrical conductance (GR). [file 13007_2023_1133_MOESM3_ESM.zip › GR_PDC6.jpg]

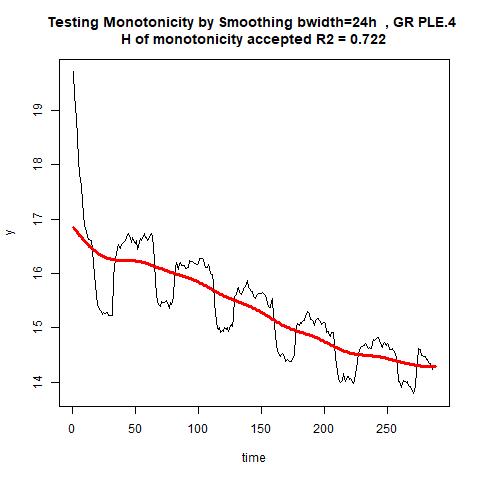

Supplement: Supplementary file 3 — Additional file 3: Monotonicity tests by nonlinear kernel smoothing with up to 24-h bandwidth for the time series of root electrical capacitance (CR), dissipation factor (DR) and electrical conductance (GR). [file 13007_2023_1133_MOESM3_ESM.zip › GR_PLE4.jpg]

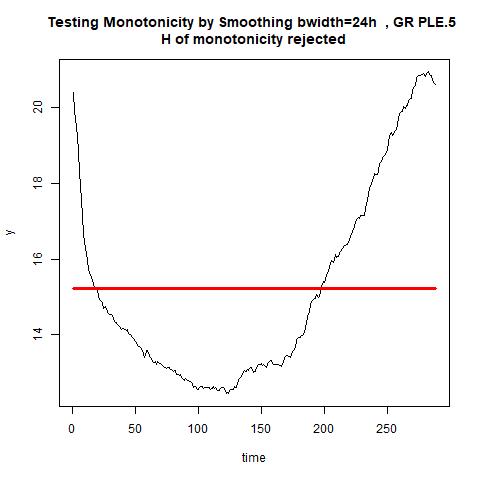

Supplement: Supplementary file 3 — Additional file 3: Monotonicity tests by nonlinear kernel smoothing with up to 24-h bandwidth for the time series of root electrical capacitance (CR), dissipation factor (DR) and electrical conductance (GR). [file 13007_2023_1133_MOESM3_ESM.zip › GR_PLE5.jpg]

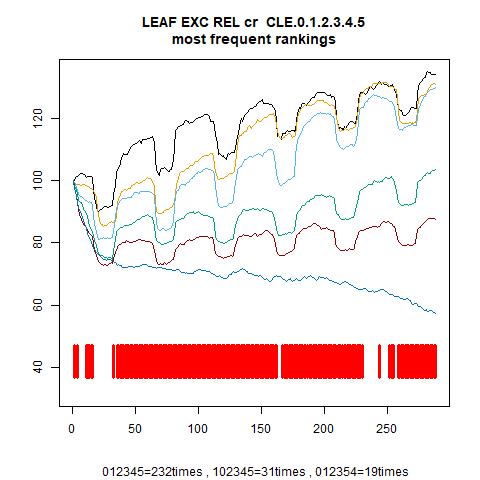

Supplement: Supplementary file 4 — Additional file 4: Order analysis for the time series of root electrical capacitance (CR), dissipation factor (DR) and electrical conductance (GR). [file 13007_2023_1133_MOESM4_ESM.zip › CR_CLE0-5.jpg]

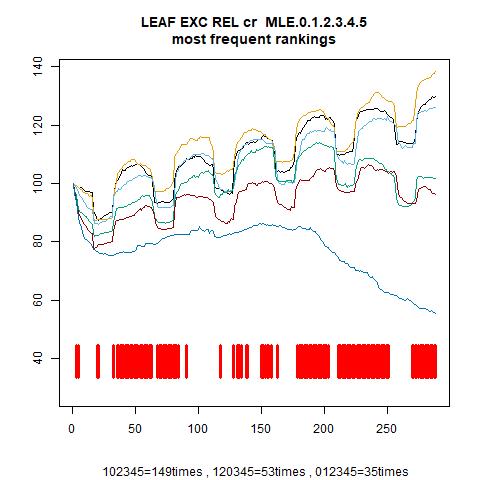

Supplement: Supplementary file 4 — Additional file 4: Order analysis for the time series of root electrical capacitance (CR), dissipation factor (DR) and electrical conductance (GR). [file 13007_2023_1133_MOESM4_ESM.zip › CR_MLE0-5.jpg]

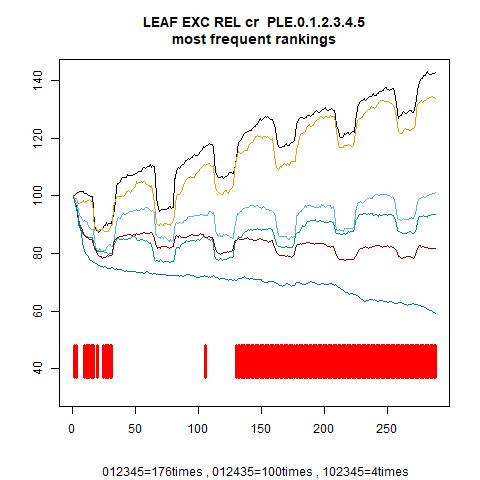

Supplement: Supplementary file 4 — Additional file 4: Order analysis for the time series of root electrical capacitance (CR), dissipation factor (DR) and electrical conductance (GR). [file 13007_2023_1133_MOESM4_ESM.zip › CR_PLE0-5.jpg]

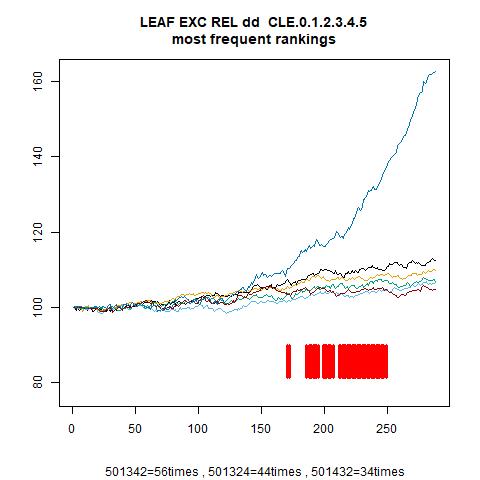

Supplement: Supplementary file 4 — Additional file 4: Order analysis for the time series of root electrical capacitance (CR), dissipation factor (DR) and electrical conductance (GR). [file 13007_2023_1133_MOESM4_ESM.zip › DR_CLE0-5.jpg]
