# Supplementary figures and images for: Monitoring of plant water uptake by measuring root dielectric properties on a fine timescale: diurnal changes and response to leaf excision (part 4 of 4)
Source: Plant Methods. 2024 Jan 9;20:5. doi: 10.1186/s13007-023-01133-8 (PMC10775601; doi:10.1186/s13007-023-01133-8)

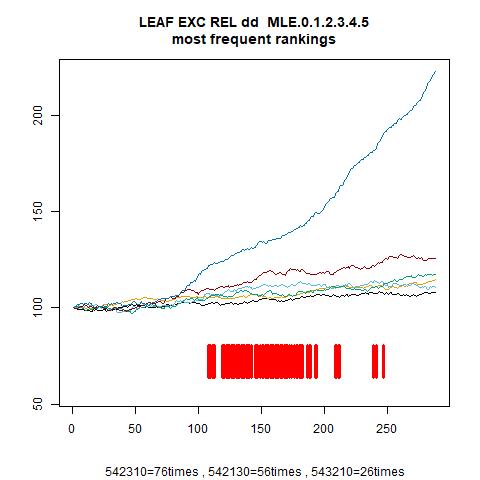

Supplement: Supplementary file 4 — Additional file 4: Order analysis for the time series of root electrical capacitance (CR), dissipation factor (DR) and electrical conductance (GR). [file 13007_2023_1133_MOESM4_ESM.zip › DR_MLE0-5.jpg]

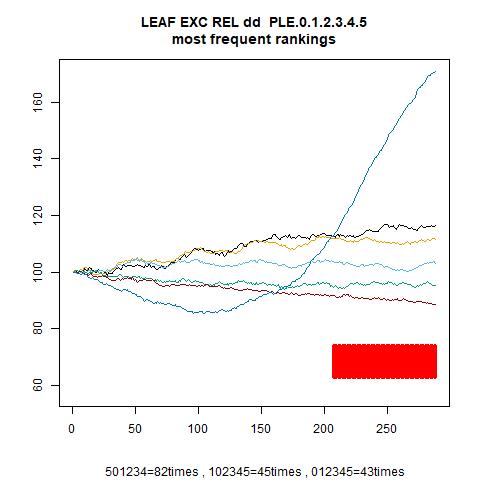

Supplement: Supplementary file 4 — Additional file 4: Order analysis for the time series of root electrical capacitance (CR), dissipation factor (DR) and electrical conductance (GR). [file 13007_2023_1133_MOESM4_ESM.zip › DR_PLE0-5.jpg]

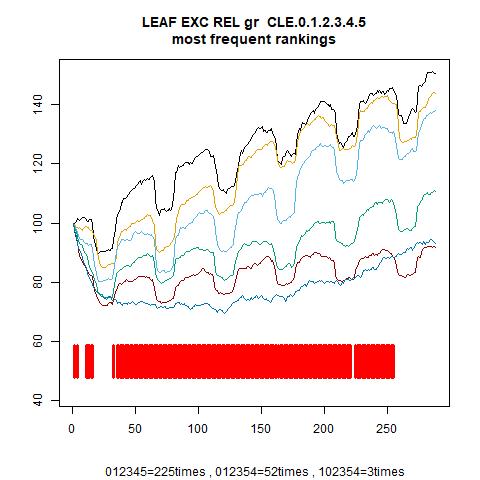

Supplement: Supplementary file 4 — Additional file 4: Order analysis for the time series of root electrical capacitance (CR), dissipation factor (DR) and electrical conductance (GR). [file 13007_2023_1133_MOESM4_ESM.zip › GR_CLE0-5.jpg]

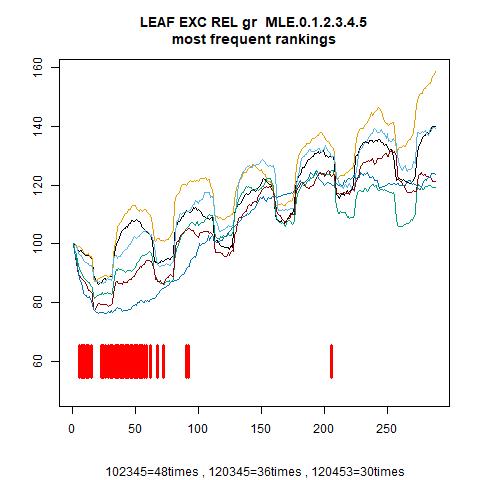

Supplement: Supplementary file 4 — Additional file 4: Order analysis for the time series of root electrical capacitance (CR), dissipation factor (DR) and electrical conductance (GR). [file 13007_2023_1133_MOESM4_ESM.zip › GR_MLE0-5.jpg]

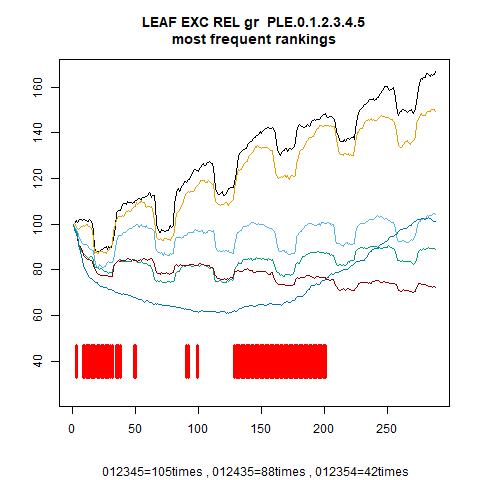

Supplement: Supplementary file 4 — Additional file 4: Order analysis for the time series of root electrical capacitance (CR), dissipation factor (DR) and electrical conductance (GR). [file 13007_2023_1133_MOESM4_ESM.zip › GR_PLE0-5.jpg]
