# Supplementary material for: Monitoring of plant water uptake by measuring root dielectric properties on a fine timescale: diurnal changes and response to leaf excision
Source: Plant Methods. 2024 Jan 9;20:5. doi: 10.1186/s13007-023-01133-8 (PMC10775601; doi:10.1186/s13007-023-01133-8)
Supplement: Supplementary file 5 — Additional file 5: Linear regressions between root electrical capacitance (CR) and stomatal conductance (gs) and between root electrical conductance (GR) and gs for plants subjected to 6-day dark period. [file 13007_2023_1133_MOESM5_ESM.pdf]

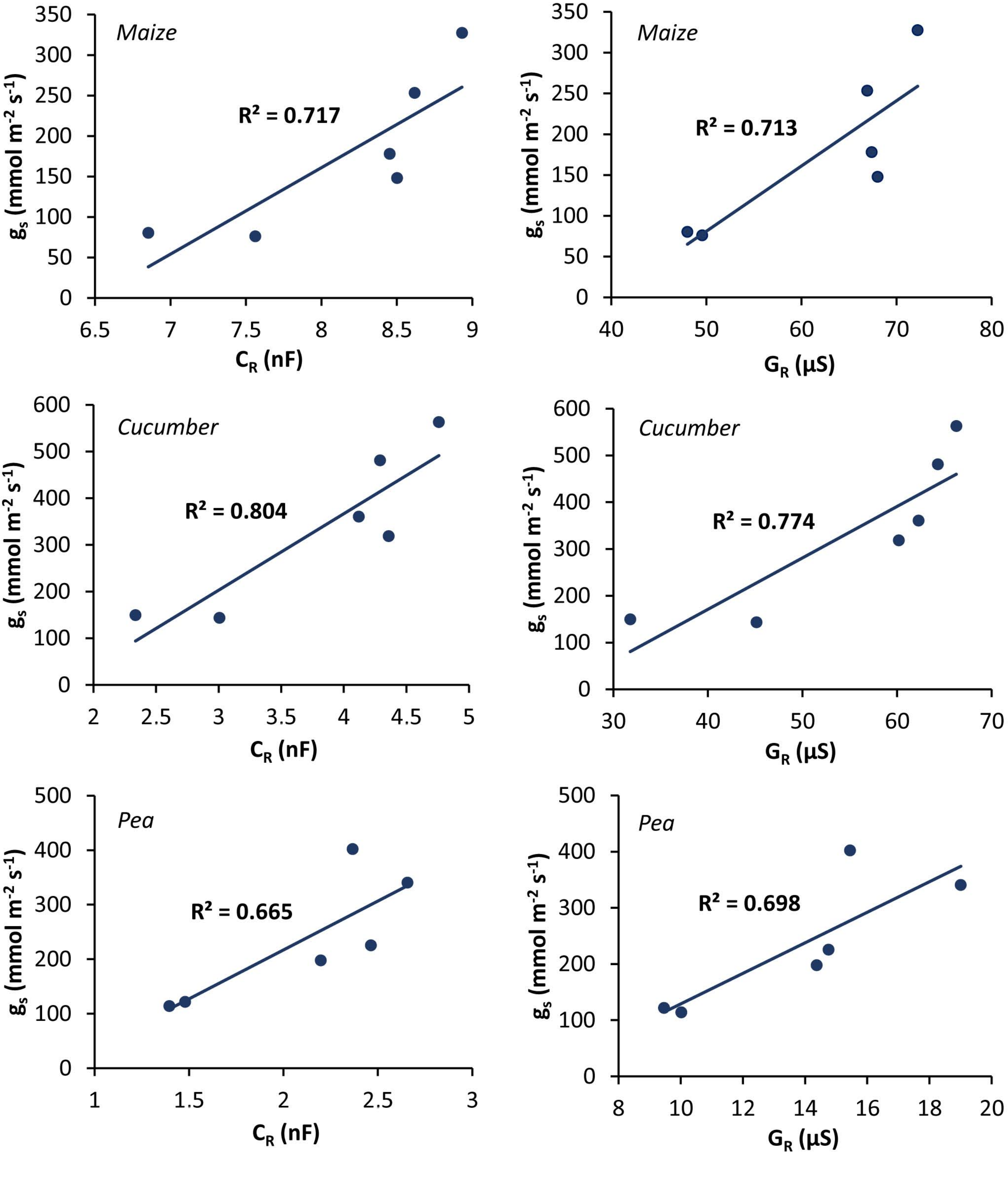

Figure S5: Linear regressions between root electrical capacitance (C_R) and stomatal conductance (g_s) and between root electrical conductance (G_R) and g_s for plants subjected to 6-day dark period.
